# Investigation of Moisture Swing Adsorbents for Direct Air Capture by Dynamic Breakthrough Studies

**DOI:** 10.1021/acssuschemeng.5c00227

**Published:** 2025-04-30

**Authors:** Yuxiang Wang, Jinsu Kim, João Marreiros, Neel Rangnekar, Yanhui Yuan, J.R. Johnson, Benjamin A. McCool, Matthew J. Realff, Ryan P. Lively

**Affiliations:** † School of Chemical & Biomolecular Engineering, Georgia Institute of Technology, 311 Ferst Drive, Atlanta, Georgia 30332, United States; ‡ Department of Petrochemical Materials, Chonnam National University, 50 Daehak-ro, Yeosu-si 59631, Republic of Korea; § Avnos, Inc., 360 Milltown Road, Bridgewater, New Jersey 08807, United States

**Keywords:** direct air capture, moisture swing adsorption, anion exchange resins, dynamic breakthrough experiments, competitive adsorption, vacuum desorption

## Abstract

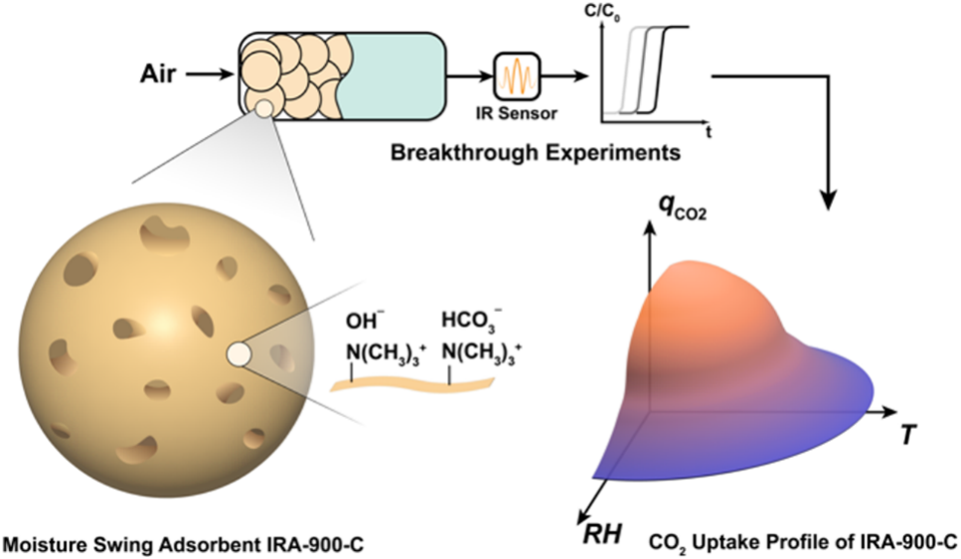

Adsorbent materials with humidity-modulated CO_2_ sorption capacities are essential for direct air capture (DAC) based on moisture swing adsorption (MSA) processes. These materials have seldom been studied in the context of dynamic breakthrough experiments despite their efficacy in providing valuable equilibrium and kinetics information on the adsorbents and their resemblance to practical processes at large scales. Herein, we performed a series of breakthrough experiments to systematically investigate the DAC properties of the MSA adsorbent IRA-900-C. Prepared from the commercially available anion exchange resin IRA-900 (chloride form), IRA-900-C exhibits a CO_2_ capacity of 1.92 mmol g^–1^ at 20% RH at 25 °C. The CO_2_ uptake capacity in IRA-900-C decreases as the environmental relative humidity (RH) increases at constant temperature. The competitive sorption behavior of CO_2_ and H_2_O is also revealed by humid CO_2_ breakthrough experiments. Breakthrough experiments with different gas velocities and particle sizes of IRA-900-C suggest that the CO_2_ adsorption kinetics in IRA-900-C is controlled by internal mass transfer resistances under DAC conditions. A theoretical maximum CO_2_ working capacity of 1.27 mmol g^–1^ can be achieved with IRA-900-C by swinging the RH from 20 to 50% RH at 25 °C along with constant purge of inert gas, and the feasibility of CO_2_ production in a vacuum is experimentally verified. This study highlights the significance of dynamic breakthrough experiments in evaluating the DAC performance of MSA sorbents and providing valuable information for the design and optimization of DAC systems enabled by moisture swing processes.

## Abstract 2

Breakthrough experiments are employed to investigate the equilibrium and kinetic CO_2_ capture properties of moisture swing adsorbents.

## 1 Introduction

Most direct air capture (DAC) technologies employed today are based on CO_2_ scrubbing by alkaline solutions, CO_2_ reactive sorption by alkali carbonates, or CO_2_ adsorption in porous materials such as zeolites, silica, aerogels, and metal–organic frameworks (MOFs).^1^ The regeneration of sorbents in these systems is essential for pseudo-steady state DAC operation, and the various regeneration processes have significant impacts on the CO_2_ purity and productivity as well as the energy consumption of the DAC processes.^2^

**Scheme 1:**
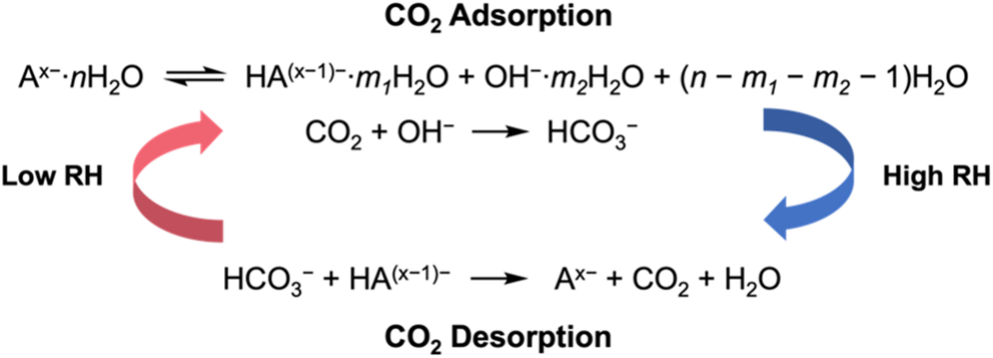
Proposed Mechanism of DAC Based on MSA^5^

DAC based on moisture swing adsorption (MSA) proposed by Lackner et al. was one of the first DAC cycles proposed. In this cycle, the CO_2_ sorbent is regenerated by exposure to high-activity water vapor or liquid water.^3,4^ Most MSA sorbents contain polymeric matrices with quaternary ammonium (QA) moieties and charge-balancing anions of weak acids. The proposed mechanism of MSA is shown in Scheme 1. When the environmental relative humidity (RH) is low, the hydrated anions A^*x*–^·*n*H_2_O hydrolyze to form OH^–^, which further reacts with CO_2_ to form HCO_3_^–^; the reaction reverses to release CO_2_ and regenerate the sorbent at high RH conditions.^5,6^ Strong base anion exchange resins based on cross-linked polystyrene are the most commonly reported MSA sorbents because they can be easily prepared from off-the-shelf resin precursors.^7,8^ In addition, aerogels and linear polymers such as PES and cellulose with QAs have recently emerged as MSA sorbents.^9−12^ In terms of the anions in MSA sorbents, a library of anions such as CO_3_^2–^, PO_4_^3–^, and B_4_O_7_^2–^ has been demonstrated to endow the sorbents with humidity-sensitive CO_2_ capturing properties, but sorbents with other anions have exhibited very limited swingable CO_2_ sorption capacity.^13,14^

To date, most prior studies have investigated the CO_2_ capture performance of MSA sorbents using the closed volumetric method. A typical evaluation system of this method is primarily composed of a sample chamber, a pump for forced convection in the system, a humidifier to regulate the system humidity, a gas injection port, and a gas composition analyzer to monitor CO_2_ and H_2_O concentrations in the system. The CO_2_ adsorption or desorption capacities of MSA sorbents are therefore determined by the initial and final CO_2_ concentrations in the system with pre-calibrated constant volume, while the sorption kinetics is inferred from the dynamic profiles of CO_2_ concentrations.^15−20^ Although it is a useful way to acquire CO_2_ sorption kinetics and changes in CO_2_ uptakes between different testing conditions using such systems, it is challenging to accurately measure H_2_O uptakes of the sorbents because humidity levels are usually maintained constant by the humidifier during the CO_2_ adsorption/desorption experiments, which could be obstacles for the accurate calculation of the energy requirements and subsequent technoeconomic analyses of MSA processes. Prior studies assumed that the weight gain of MSA sorbents during CO_2_ desorption steps was mainly attributed to H_2_O adsorption because the amount of desorbed CO_2_ was negligible.^16^ This hypothesis may not apply to MSA sorbents with high CO_2_ uptake and significant levels of hydrophobicity. In addition, the closed volumetric method does not offer information on column dynamics of the CO_2_ capture bed, which reflects the sorbents’ sorption properties (both thermodynamics and kinetics), dispersion, and thermal behavior of the packed bed. Such information is critical for scaling up MSA technologies.

Dynamic breakthrough experiments, a category of open volumetric methods, have been widely employed to study the performance of sorbents for gas-phase chemical separation applications such as CO_2_ capture, hydrocarbon separations, and air separations.^21^ In a typical breakthrough experiment, a gas stream is fed into a packed bed continuously, and the gas compositions and flow rates at the outlet of the packed bed are recorded to generate breakthrough curves. The uptakes of the sorbates can be calculated from their corresponding breakthrough curves, and the sorption kinetic parameters can be derived by fitting the breakthrough curves with computational breakthrough models that take all factors (e.g., sorbent properties, axial dispersion, thermal effects, etc.) into consideration. As breakthrough experiments resemble the sorption steps of practical separation processes, they provide valuable connections between bench-scale lab experiments and pilot or commercial chemical separation processes. Nevertheless, very few prior studies have investigated MSA sorbents using dynamic breakthrough experiments under DAC-relevant conditions.^16,22,23^

In this work, we present the systematic evaluation of an MSA sorbent based on off-the-shelf resin IRA-900 using dynamic breakthrough experiments. CO_2_ uptake capacities in the MSA sorbent were measured under different temperatures and humidities. In addition to the CO_2_ adsorption and desorption properties, the H_2_O coadsorption capacity of the sorbent during CO_2_ capture experiments was also carefully studied. We envision that this work will be a useful guideline for the evaluation of MSA sorbents with breakthrough experiments.

## 2 Materials and Methods

### 2.1 Chemicals

Amberlite IRA-900 chloride form and KHCO_3_ (ACS reagent, 99.7%) were purchased from Sigma-Aldrich. K_2_CO_3_ (ACS reagent, 99.0%) was purchased from VWR Chemicals BDH. DI water was obtained from an Elga PureLab Option S7 water purification system. Cylinders of N_2_ (99.995%), Ar (99.995%), and N_2_ with different concentrations of CO_2_ (400 ppm, 1000 ppm, 1%) were purchased from Airgas.

### 2.2 Characterizations

Elemental analysis was conducted by Atlantic Microlab. SEM. Scanning electron microscopy (SEM) images were obtained with a Hitachi SU8010. Before imaging, samples were sputtered with a Quorum Q-150T ES for 40 s. Unary water vapor adsorption isotherms were obtained with a TA Instruments VTI-SA+ after the *in situ* activation of samples by N_2_ purging at 35 °C for 12 h.

### 2.3 Preparation of IRA-900-C

IRA-900-C was synthesized via a series of ion exchange processes. Typically, about 1 g of IRA-900 beads in their chloride form were soaked in 40 mL of 1 M KHCO_3_ aqueous solution with gentle shaking. The aqueous supernatant was decanted after 24 h, and the beads were washed by DI water until the washing liquid was neutral (pH = 7). The beads were redispersed in 40 mL of fresh 1 M KHCO_3_ aqueous solution for another round of ion exchange, which was repeated until no Cl^–^ could be detected by Ag^+^ titration with Mohr’s method. The beads were washed by DI water thoroughly before drying in the fume hood at room temperature (23 °C).

### 2.4 Dynamic Breakthrough Experiments

**Scheme 2:**
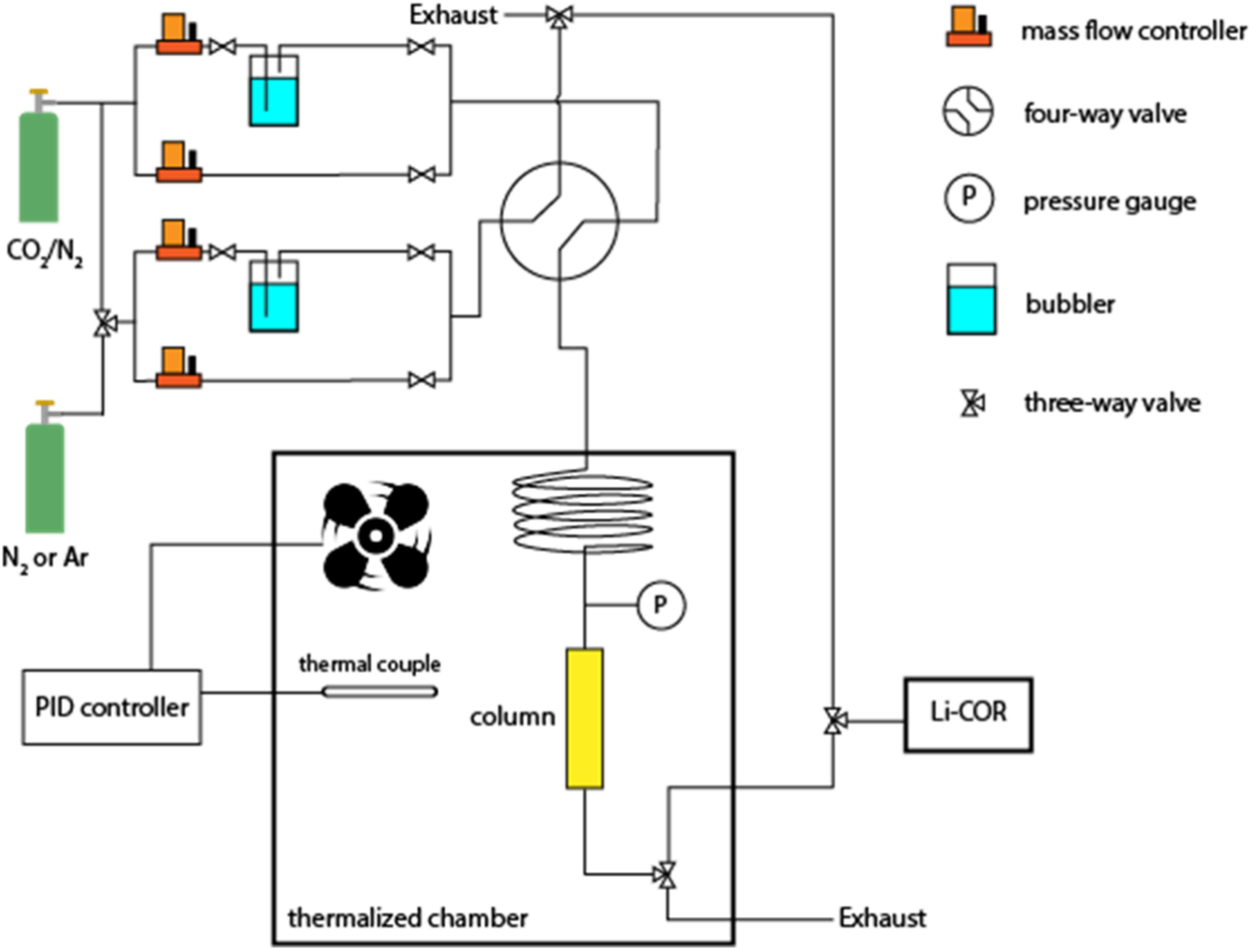
A Scheme of the Breakthrough Setup (Open Volumetric) Used for the Evaluation of MSA Adsorbents The whole setup is placed in a thermalized chamber with temperature control for measuring CO_2_ uptakes at different relative humidities (RHs) at 35 °C to prevent water condensation in the system.

A schematic illustration of the setup for breakthrough experiments is shown in Scheme 2. The mass flow controllers (MFCs) in the setup were purchased from Alicat Scientific. The CO_2_ and water vapor concentrations at the outlet of the adsorption bed were measured by an infrared gas analyzer LI-850-1 produced by LI-COR Environmental, and the relative humidities (RH) in the system were cross-checked by a RH and temperature probe HMP8 of Vasaila. Humid gas streams were generated by flowing N_2_ or CO_2_/N_2_ through the customized bubblers that can withstand buildup pressures of the packed bed of IRA-900-C, and the relative humidities were controlled by adjusting the flow rates of dry and humid streams. The whole setup was placed in a thermalized chamber programmed at specific temperatures to prevent water condensation in the system.

A typical breakthrough experiment is described as follows. IRA-900-C resins after anion exchange (110–300 mg) were packed into a 1/4″ stainless steel column whose both ends were blocked by stainless steel meshes to prevent the resin particles from leaving the bed during the breakthrough experiment. The bed was activated by humid inert gas (200 sccm Ar or N_2_) with an RH greater than 85% at 25 °C overnight until the CO_2_ concentrations detected at the outlet of the bed were stable and less than 3 ppm. A stabilized stream of 400 ppm CO_2_ balanced by N_2_ (200 sccm) with a desirable RH was then introduced into the bed at 25 °C, and the concentrations of CO_2_ and H_2_O at the outlet of the bed were recorded. The bed was determined to be saturated by the feeding of CO_2_ when the outlet CO_2_ concentration was equal to the feeding concentration. The bed was regenerated under humid inert gas (200 sccm Ar or N_2_) with an RH greater than 85% at 25 °C overnight before being purged by a flow of dry inert gas (500 sccm N_2_) to determine the dry mass of sorbent used in the breakthrough experiment.

### 2.5 CO_2_ Desorption under Vacuum

**Scheme 3:**
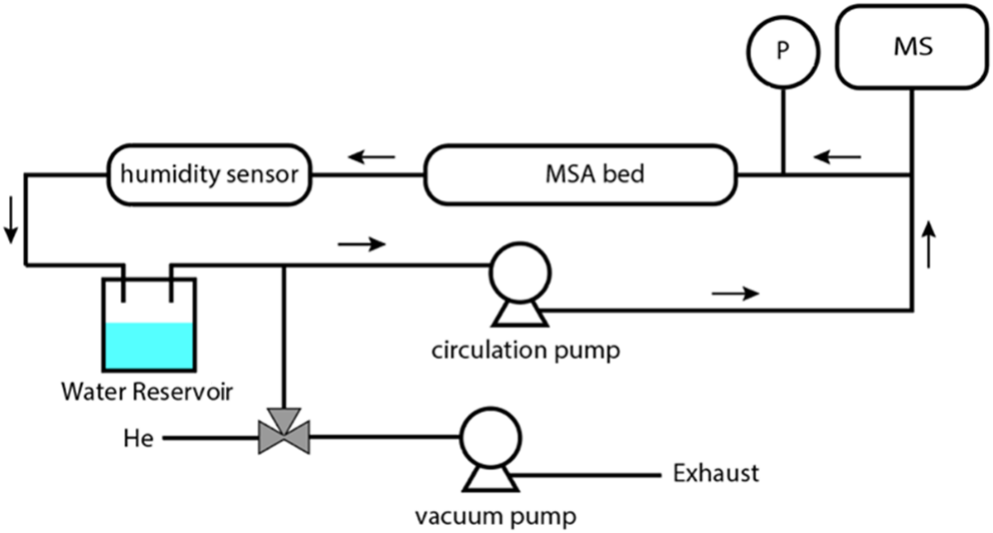
A Scheme of the Experimental Setup (Closed Volumetric) for the Regeneration of MSA Adsorbents by Water Vapor in Vacuum Arrows indicate the flowing direction of water vapor when the circulation pump is in operation.

A schematic illustration of the closed volumetric setup for CO_2_ desorption in a vacuum under forced convection is shown in Scheme 3. In a typical experiment, a diaphragm pump was kept running to force convection within the system. Water in the reservoir was first frozen before evacuating the system for 30 s with a Pfeiffer DUO 2.5 dual-stage rotary vane vacuum pump. The vacuum degree was about −95 kPa. The vacuum pump was then isolated, and the ice in the water reservoir was thawed to release dissolved gases. The freeze–pump–thaw cycles were repeated three times before warming up the water reservoir to 30 °C to achieve target RH levels in the system for sorbent regeneration (while other parts of the system remained at ambient temperature). The water partial pressure of the setup was monitored by Vasaila HMP8, and the gas compositions in the system were probed by a mass spectrometer Pfeiffer Omnistar GSD 320.

## 3 Results and Discussion

### 3.1 Structural Characterization of the MSA Adsorbent

IRA-900 is an anion exchange resin based on macroporous cross-linked copolymers of styrene and divinylbenzene. Quaternary ammonium (QA) sites are covalently grafted onto the polymer backbones of IRA-900, and the positive charges are balanced by chloride anions that can be substituted by other anions via anion exchange (Scheme 1). There have been several prior works that studied the CO_2_ capture performance of IRA-900 derivatives. He et al. reported that IRA-900 with triazine anions exhibits a CO_2_ uptake as high as 4.02 mmol g^–1^ at 0.15 bar and 25 °C, outperforming IRA-900 with other anions under the same conditions.^7^ Wang et al. studied IRA-900 along with other anion exchange resins with different basicity and revealed that the form of QA sites determine CO_2_ uptake capacities, while the textural properties affect CO_2_ sorption kinetics under conditions relevant to direct air capture of CO_2_ (DAC).^24^ A recent study by Hegarty et al. screened a variety of anions of IRA-900 and revealed that IRA-900 with pyrophosphate possesses the highest CO_2_ swing capacity per ammonium site when the relative humidity (RH) in the closed chamber was increased from 20 to 65%.^25^

**Figure 1:**
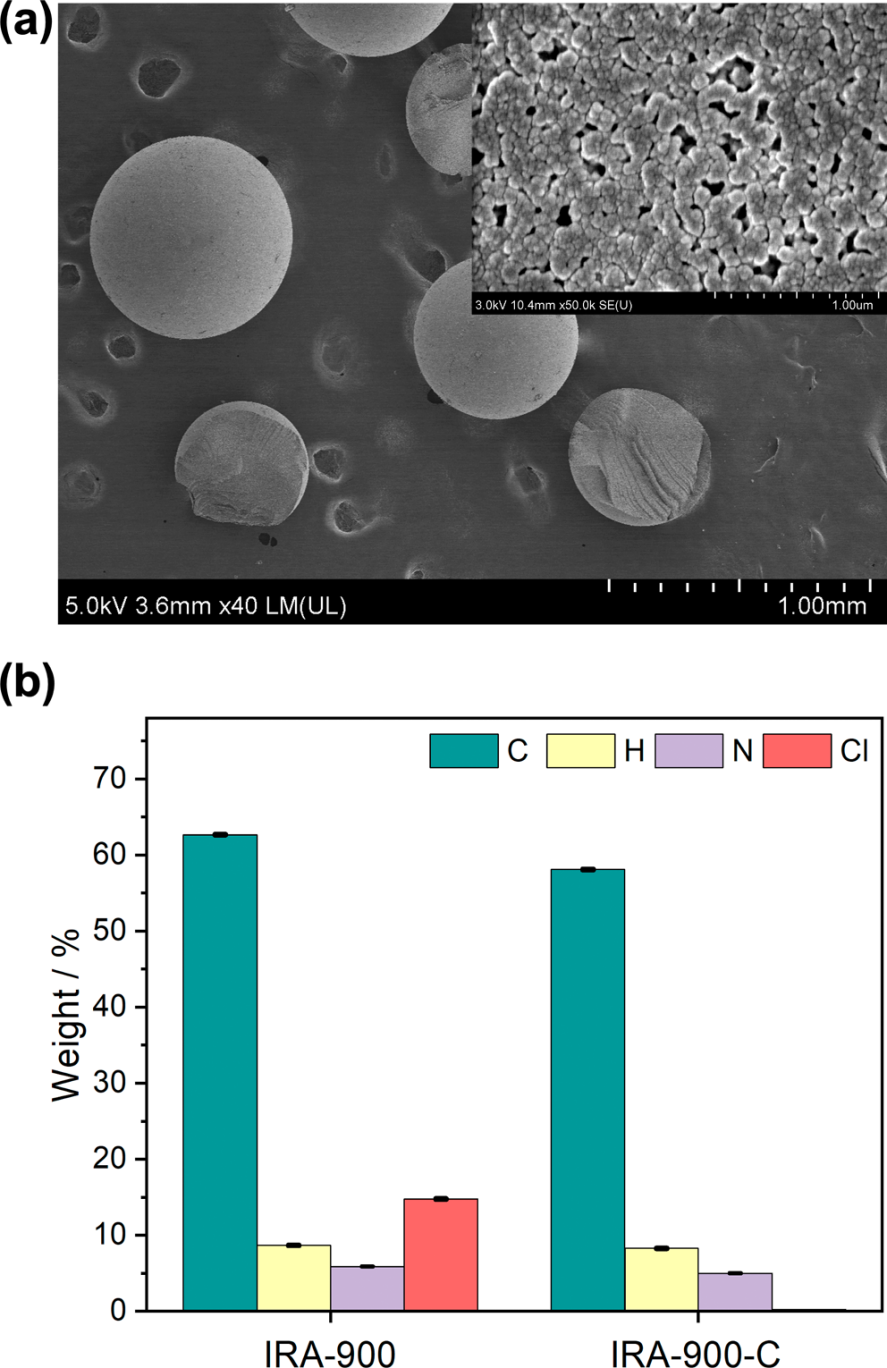
(a) FE-SEM images of IRA-900-C. The inset shows the macroporous surface texture of IRA-900-C. (b) Elemental compositions of IRA-900 and IRA-900-C.

In this work, rather than comparing the DAC performance of different IRA-900 resins, IRA-900 balanced by HCO_3_^–^/CO_3_^2–^ was selected as the model material to develop the evaluation system of MSA sorbents based on dynamic breakthrough experiments. The anion exchange experiment was initially performed by immersing IRA-900 beads in a 0.5 M K_2_CO_3_ aqueous solution. However, the resin dispersion emitted a fishy odor after anion exchange, hinting at the decomposition of IRA-900 and the release of trimethylamine under high pH conditions, even though the extent of decomposition might be very low. Subsequent anion exchange experiments were carried out in 1 M KHCO_3_ aqueous solution, during which the fishy odor was not noticed. The anion-exchanged IRA-900 (denoted as IRA-900-C) was then dried in a fume hood at room temperature before being packed into a column for breakthrough experiments. The macroporous nature of IRA-900 was preserved after anion exchange, as examined by scanning electron microscope (SEM), but the surface of IRA-900-C was smoother, probably due to particle attrition during the anion exchange process (Figures 1a and S1). Only trace amounts of chlorine could be detected in IRA-900-C (Figure 1b and Table S1), which, along with titration tests, suggests almost complete anion exchange from Cl^–^ to HCO_3_^–^. The amount of QA moieties in IRA-900 was calculated to be 4.18 mmol g^–1^ based on the N content of the resin determined by elemental analysis. Therefore, a maximum of 2.09 mmol g^–1^ OH^–^ can be generated in the resin via HCO_3_^–^ hydrolysis under low RH conditions according to Scheme 1.^4^ It should be noted that it is challenging to obtain fully dehydrated IRA-900-C due to its tendency of decomposition in the dry state. Therefore, the residual water molecules in the resins, though minimal, will lead to a slightly overestimated dry mass of the resin and an underestimated N content and QA concentrations in the resin. The average diameter of IRA-900-C beads is 614 μm, and the particle size distribution is shown in Figure S2.

### 3.2 CO_2_ Adsorption Capacities of IRA-900-C

**Figure 2:**
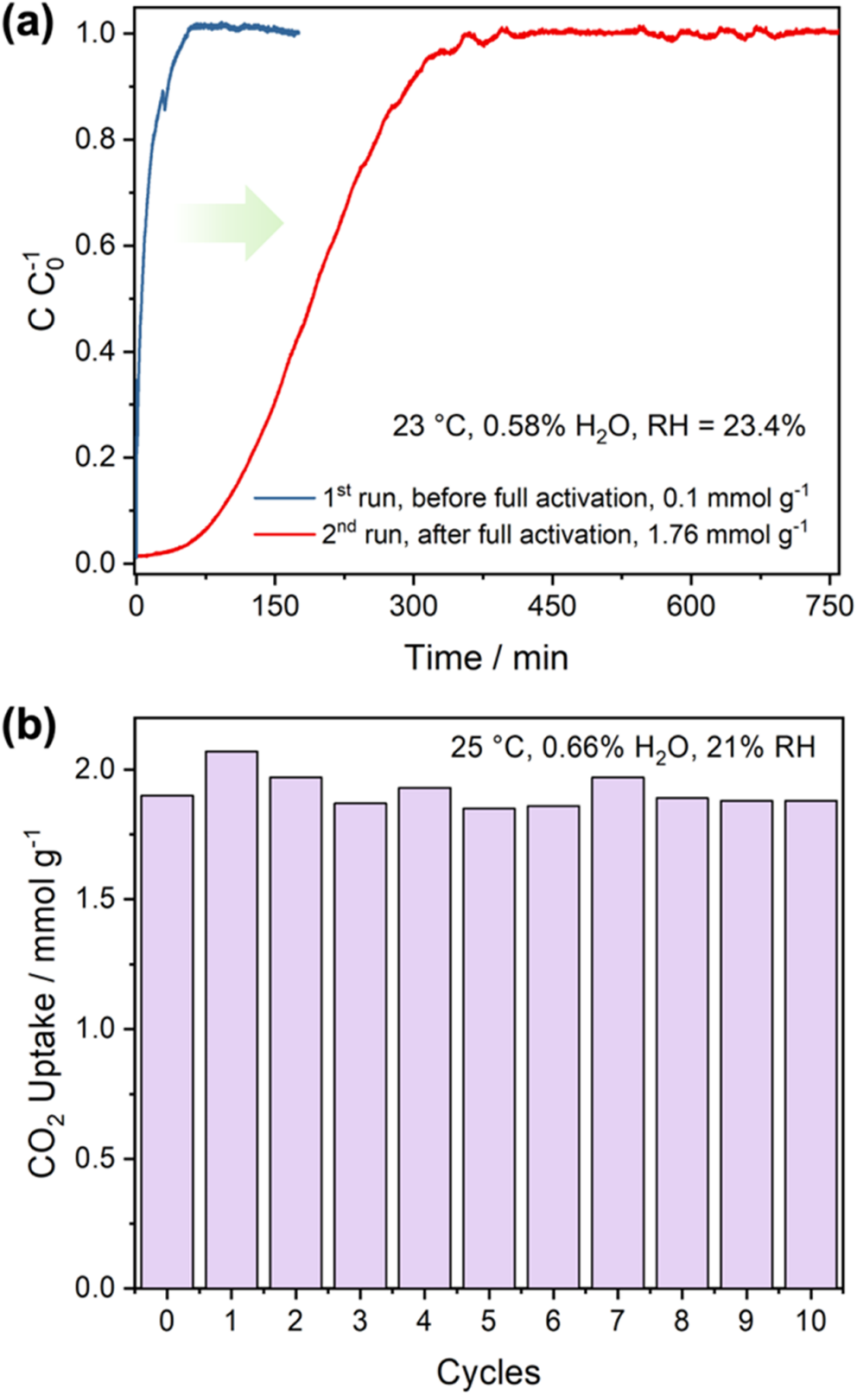
(a) CO_2_ breakthrough curves of the IRA-900-C packed bed before and after full activation. These curves were obtained using 400 ppm CO_2_/balance N_2_ with 0.58 mol % H_2_O (23.4% RH) at 23 °C. The dry mass of IRA-900-C in the bed was 357.0 mg. (b) CO_2_ adsorption capacities in IRA-900-C over 10 cycles of breakthrough experiments using 400 ppm CO_2_/balance N_2_ with 0.66 mol % H_2_O (21% RH) at 25 °C.

Figure 2a shows the 400 ppm CO_2_ breakthrough curves of the initial two breakthrough experiments using a packed bed of IRA-900-C. The column was purged by dry N_2_ at room temperature until no CO_2_ was observed at the column outlet according to the CO_2_ and H_2_O IR analyzer before the first breakthrough experiment. Although this is a mild activation method, it is sufficient to desorb physisorbed CO_2_ from the packed bed if there is any while still retaining chemisorbed CO_2_ in the form of HCO_3_^–^ (which should require moisture to desorb). Interestingly, only a negligible amount of CO_2_ (0.1 mmol g^–1^) was adsorbed by the column at 23 °C and 23.4% relative humidity (RH) after the dry inert gas activation step, indicating the ultrasmall population of CO_2_ physisorption sites in IRA-900-C. As prior works have reported the activation of MSA sorbents under high RH atmosphere, the column was subsequently purged by humid Ar with a RH of 80% at 23 °C overnight before the second breakthrough experiment. CO_2_ uptake in the column was dramatically increased to 1.76 mmol g^–1^ after the activation step with humid Ar, which underscores the moisture swing properties of IRA-900-C. Interestingly, when dry 400 ppm CO_2_ is used for the breakthrough experiment, the CO_2_ uptake kinetics was slower as suggested by the more dispersed breakthrough profile, and the CO_2_ uptake was much lower (0.97 mmol g^–1^) after 6 h of the experiment even though the bed was fully activated in humid Ar (Figure S3b, 3rd run test). Further overnight purging by dry Ar did not recover any CO_2_ uptake capacity (Figure S3b, 4th run test). These experiments demonstrate the essential role of water vapor in both CO_2_ adsorption and desorption in IRA-900-C. Water vapor coadsorbed in IRA-900-C in the CO_2_ capture step helps dilate the resin and open the CO_2_ diffusion pathway toward otherwise inaccessible/hard-to-access sorption sites, and water vapor also drives the release of CO_2_ and converts HCO_3_^–^ to CO_3_^2–^ in the desorption step for subsequent CO_2_ adsorption cycles. Encouraged by the MSA phenomena observed by breakthrough experiments, the anion exchange experiments were repeated, and a new batch of IRA-900-C was used for repetitive breakthrough experiments using 400 ppm CO_2_ at 25 °C and 21% RH. The IRA-900-C column was purged by humid Ar with 84 ± 1% RH at 25 °C overnight after each adsorption experiment. The resin exhibited consistent CO_2_ uptake capacities over 10 cycles with an average CO_2_ uptake capacity of 1.92 mmol g^–1^ at 400 ppm CO_2_ (Figures 2b and S4). As the majority of CO_2_ is captured by the reaction between CO_2_ and OH^–^, more than 90% of OH^–^ in IRA-900-C is utilized for CO_2_ capture based on the QA concentration in IRA-900 and the stoichiometry between QA and OH^–^ (two QA sites are balanced by one HCO_3_^–^ and one OH^–^). The utilization efficiency of OH^–^ under this circumstance is significantly greater than the amine efficiency of DAC solid adsorbents, which rarely exceeds 25%.^26,27^

The CO_2_ uptake in IRA-900-C is higher than what has been reported in the literature earlier (∼0.76 mmol g^–1^),^13,24^ which might be attributed to the different methodologies of determining CO_2_ uptakes used in this work and prior studies. Most of the prior works on MSA adsorbents calculated CO_2_ uptakes based on the concentration changes of CO_2_ in a system with constant volume as a result of the changes in RH. These uptakes are relative CO_2_ uptakes between two humidity levels or CO_2_ working capacity of the sorbent, and they will converge to the true CO_2_ uptake at the lower RH condition when the absolute CO_2_ uptake in the sorbent under regeneration conditions approaches zero. In contrast, as high RH inert gas purging was adopted as the regeneration step to maximize both chemical (water activity) and physical (CO_2_ partial pressure) driving forces for CO_2_ desorption in this work, the CO_2_ uptakes determined from breakthrough experiments most likely represent the absolute CO_2_ uptakes in the IRA-900-C.

**Figure 3:**
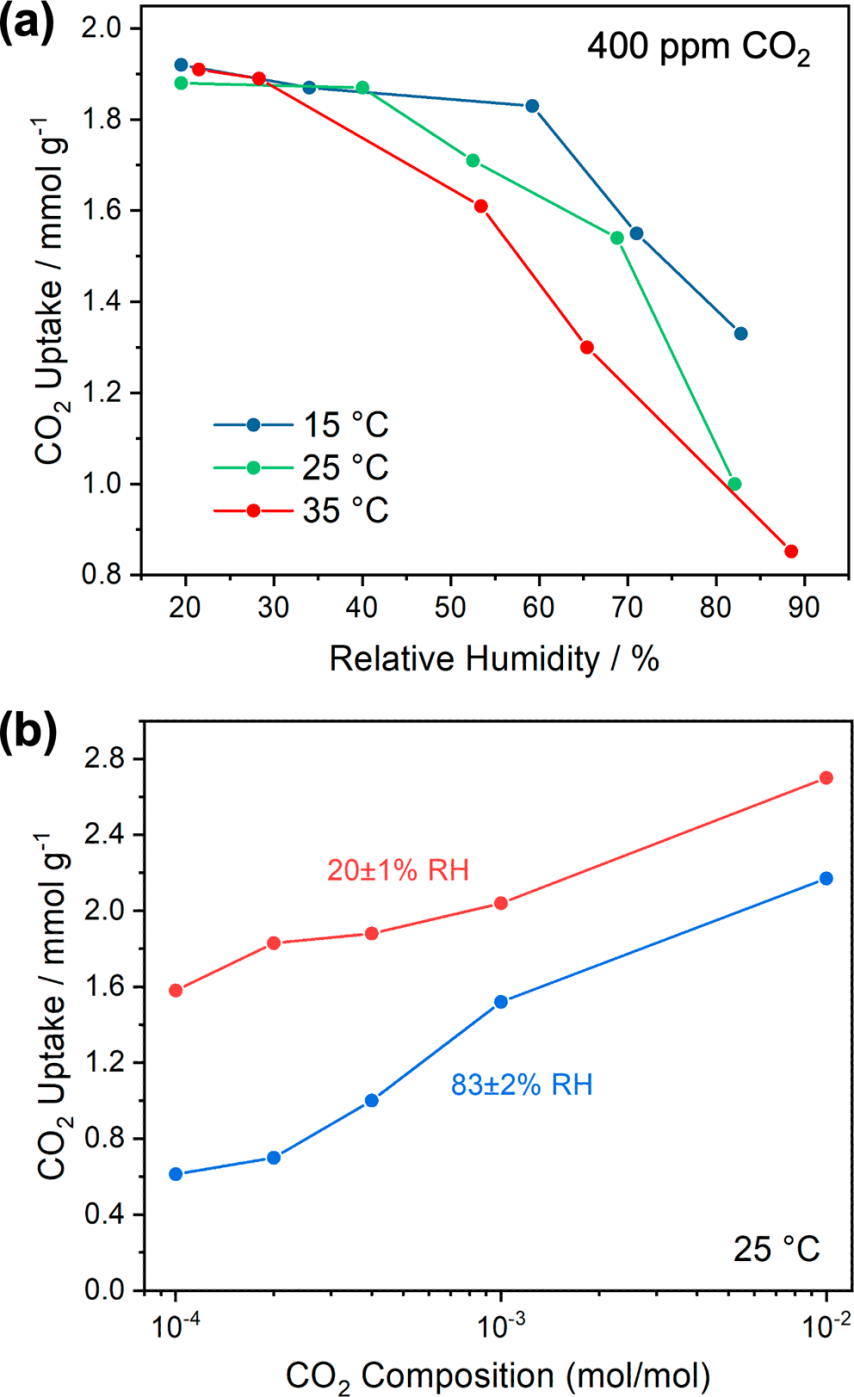
(a) CO_2_ adsorption capacities in IRA-900-C at a CO_2_ composition of 400 ppm (total pressure 1 bar) as a function of RHs at 15, 25, and 35 °C. (b) CO_2_ adsorption capacities at different CO_2_ compositions under dry (20.5 ± 1%) and humid (83 ± 1%) conditions at 1 bar and 25 °C.

A series of breakthrough experiments using 400 ppm CO_2_ with different water content were carried out at different temperatures to investigate the effects of moisture and temperature. The CO_2_ uptakes obtained from these breakthrough experiments were plotted against different humidity levels and are shown in Figures 3a and S5. CO_2_ uptakes are generally higher at lower temperatures under similar RH conditions, which reflects the exothermic nature of the CO_2_ adsorption. Interestingly, IRA-900-C possesses comparable CO_2_ uptakes (∼1.9 mmol g^–1^) under relatively dry conditions (RH < 30%) at all investigated temperatures, suggesting a nearly full coverage of CO_2_ sorption sites by 400 ppm CO_2_ even at 35 °C. CO_2_ uptakes only decrease slightly at the low RH region but suffer a greater decline when RH increases, and the effects of RH are more pronounced when temperatures are higher. The difference between CO_2_ uptakes at 21 and 86.5% RH at 25 °C is about 0.88 mmol g^–1^, which is similar to the literature value determined in a constant-volume system.^24^ The results in Figure 3a clearly showcase the dependence of CO_2_ uptakes on RH at different temperatures, which are consistent with what has been reported in similar MSA sorbents based on ion exchange resins. These results also suggest that a greater CO_2_ productivity can be achieved by spending additional energy input to slightly increase desorption temperatures in an MSA process. A recent modeling study suggests that heating the MSA sorbents during the desorption step can improve the thermodynamic efficiency of the overall DAC process.^28^

In addition to the significant impacts of RH on the CO_2_ uptake capacities, the hydration degree of resins at the beginning of breakthrough experiments also affects the CO_2_ uptake capacities and kinetics, the relationship of which has been relatively less investigated compared to the relationships between RH and CO_2_ uptakes. As shown in Figure S6a, the CO_2_ concentration at the column outlet increased continuously from the beginning of the breakthrough experiment using a column pre-dried to the humid level of the feeding gas after column regeneration under a high RH atmosphere. In comparison, the CO_2_ breakthrough curve of a column without the pre-drying step shows a stepwise profile with a similar early CO_2_ breakthrough moment as shown in Figure S6b. Both experiments afford comparable CO_2_ uptake capacities under equilibrium, despite the different shapes of breakthrough curves. Theoretically, the pre-dried bed of IRA-900-C should have more OH^–^ sites for CO_2_ adsorption due to the hydrolysis of CO_3_^2–^ at low RH conditions and hence afford a sharper CO_2_ breakthrough profile than that of the column without the pretreatment, assuming that the mass transfer rates of CO_2_ are comparable in both columns. The discrepancy between the experimental observation and theoretical analysis suggests a slower CO_2_ mass transfer kinetics in the pre-dried column, which is likely due to the narrower diffusion pathways for CO_2_ in the less-dilated resin sorbents. In addition, the different CO_2_ uptake kinetics could be attributed to the greater diffusion coefficients of all ionic species at higher RHs due to the more connected water network in the pores. The interactions between the QA sites and negatively charged species are also interrupted by water in the vicinity, according to recent simulation findings.^29,30^ When the resin was purged by dry N_2_ overnight before the breakthrough experiment, the level of uptake of CO_2_ in this resin was even lower than those of resins with greater hydration degrees before the breakthrough experiments (Figure S6c). We surmise that the accessibility of quaternary ammonium sites is greatly inhibited in the severely dehydrated IRA-900-C. As the polystyrene backbone of IRA-900-C is hydrophobic, rehydrating IRA-900-C with a moderate level of humidity (the humidity used for the breakthrough experiment) may be insufficient to release all active sites trapped in hydrophobic domains, leading to the compromised CO_2_ uptake capacity. In comparison, when IRA-900-C is fully hydrated after exposure to 85% RH N_2_, the resin is fully swollen, and the active sites remain accessible for CO_2_ sorption even though H_2_O desorbs from IRA-900-C gradually during the breakthrough experiment.

Unary CO_2_ uptake isotherms of common CO_2_ adsorbents have been routinely measured by using commercial gas sorption analyzers. Nevertheless, the coadsorption capacities of CO_2_ in the presence of water, which are important metrics for DAC sorbents, especially those for MSA processes, have been investigated less. Breakthrough experiments of gas streams with various CO_2_ compositions were conducted to measure CO_2_ uptake isotherms of IRA-900-C at dry (20.5 ± 1% RH) and humid (83 ± 1% RH) conditions at 25 °C, and the results are presented in Figure 3b. IRA-900-C loses CO_2_ uptake significantly as the CO_2_ composition decreases under the high RH condition. In comparison, CO_2_ uptake only drops about 15% as the CO_2_ composition decreases from 400 to 100 ppm under the low RH condition. CO_2_ increases substantially when the composition of CO_2_ increases up to 1%, which is attributed to the physisorption of CO_2_ in the highly polar pore environment of IRA-900-C. CO_2_ uptakes at high CO_2_ compositions (1000 and 1%) also exhibit a similar dependence on relative humidity as the CO_2_ uptakes at 400 ppm (Figure S7). It is worth noting that the CO_2_ uptake at 1% CO_2_ composition and 83% RH at 25 °C (2.17 mmol g^–1^) is greater than the uptake at 400 ppm CO_2_ and 20% RH (1.92 mmol g^–1^) at the same temperature. As a result, only a small fraction of captured CO_2_ could be desorbed from IRA-900-C under humid conditions (e.g., 83% RH) at 25 °C as IRA-900-C reaches a new equilibrium with the desorbed CO_2_ in the free volume of a closed system, even though the CO_2_ partial pressure is still relatively low. Higher desorption temperature or continuous removal of desorbed CO_2_ out of the system to maintain a low CO_2_ partial pressure would be necessary to achieve meaningful CO_2_ desorption. Interestingly, the CO_2_ uptake at 1% CO_2_ composition at 20% RH, 35 °C in IRA-900-C is even higher than the uptake at the same CO_2_ partial pressure and 25 °C (Figure S8). Although this abnormal observation appears to violate the thermodynamics of adsorption, it has been reported for DAC solid sorbents based on supported amines, and the observed lower CO_2_ uptakes at relatively low temperatures are attributed to CO_2_ mass transfer limitations and insufficient equilibrium time.^31^ We speculate that more available CO_2_ sorption sites and faster diffusion kinetics of gas/ionic species synergistically contribute to the observed enhancement of the CO_2_ uptake capacity at 35 °C.

### 3.3 Analysis of CO_2_ Mass Transfer Resistances in IRA-900-C

**Figure 4:**
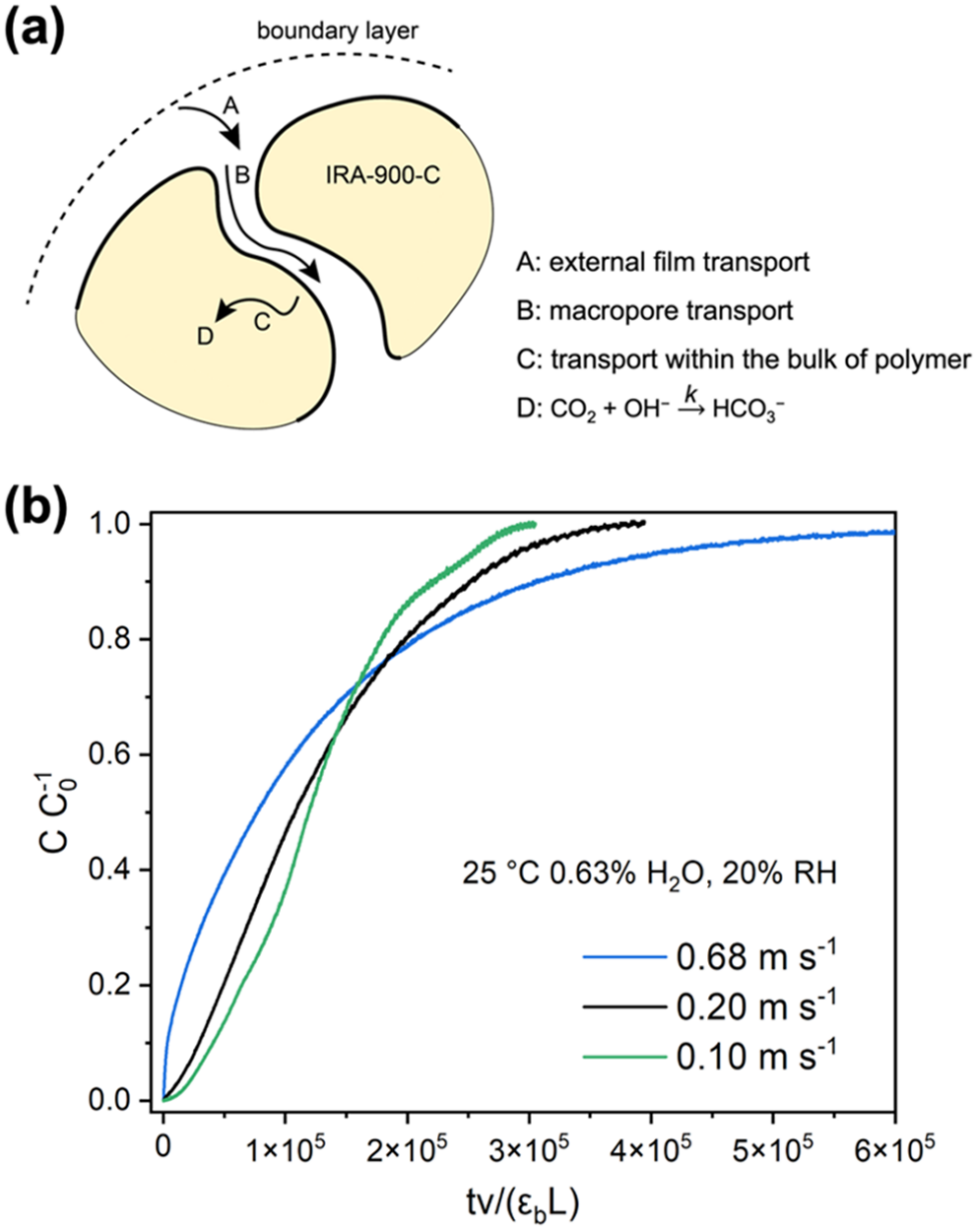
(a) Schematic illustration of CO_2_ transport from the gas bulk to the sorption sites where CO_2_ is converted to HCO_3_^–^. (b) CO_2_ breakthrough curves of the IRA-900-C packed bed using 400 ppm CO_2_/balance N_2_ with 0.63 mol % H_2_O (20% RH) and different linear velocities 25 °C. The IRA-900-C bed was pre-equilibrated with N_2_ carrying 0.63 mol % H_2_O (20% RH) at 25 °C before the breakthrough experiment. The breakthrough curves are plotted with dimensionless breakthrough time, which is converted from the actual breakthrough time *t* (s) based on the linear velocity of the feeding gas *v* (m s^–1^), bed porosity ε_*b*_, and the bed length *L* (m).

The mass transfer information on different gas components in addition to their pseudo-equilibrium uptake capacities is crucial for the development of optimal DAC processes based on MSA sorbents with high CO_2_ productivity and low costs.^32^ CO_2_ from the bulk flow needs to travel through the thin film boundary layer, the macropores, and the polymer bulk of IRA-900-C before OH^–^ is combined to form HCO_3_^–^, as shown in Figure 4a. Prior works have built models to derive the mass transfer resistances and gas diffusivities of CO_2_ or H_2_O in MSA sorbents based on CO_2_ concentration profiles or mass changes of the sorbents in the closed system.^15,19,33^ In this work, a series of breakthrough experiments were carried out to identify the primary mass transfer resistance in IRA-900-C under conditions related to DAC. First, streams of 400 ppm CO_2_ containing 0.63 mol % H_2_O (20% RH) at different velocities were fed into the bed of IRA-900-C, which was pre-dried at the humidity level of the CO_2_ streams at 25 °C. The CO_2_ breakthrough curves of these experiments were plotted in Figure 4b where the *x* axes of the breakthrough curves were normalized according to the gas velocities, the bed porosity, and bed length. Although increasing the flow rates of CO_2_ streams do not affect the pseudo-equilibrium CO_2_ uptake capacities in IRA-900-C, faster gas velocities led to earlier CO_2_ breakthrough moments and wider mass transfer zones. As we estimate that adsorption heat and axial dispersion have only minor contributions to the broadening of the mass transfer zone (detailed discussion in the Supporting Information), we surmise based on these results that the internal mass transfer processes, namely, CO_2_ diffusion in the macropores of the resin and CO_2_ diffusion in the bulk of the cross-linked polystyrene frameworks, or the reaction kinetics of bicarbonate formation, control the rate of CO_2_ adsorption.

Prior work has observed faster CO_2_ sorption kinetics in IRA-900 with smaller particle sizes in a constant-volume sorption chamber.^24^ To understand how particle sizes affect the CO_2_ sorption kinetics in IRA-900-C under dynamic conditions, IRA-900 was ground by ball milling at cryogenic temperatures before being subjected to anion exchange and particle sieving for breakthrough experiments. The ground IRA-900-C sample is denoted as IRA-900-C-g. As shown in Figure S9a, the ground resin shows a particle size distribution of 10-100 μm, which is about 1/10 of the size of the IRA-900-C beads. SEM revealed the retention of the resin’s macropores after cryogrinding (Figure S9b). IRA-900-C-g possesses a comparable CO_2_ uptake capacity to that of IRA-900-C beads but different CO_2_ column dynamics under the same RH and temperature conditions (Figure S9c). The CO_2_ breakthrough curve of the IRA-900-C-g column showed a sharper profile at the beginning (C C_0_^–1^ < 0.5) followed by a gradual-slope profile at the later stage of the breakthrough experiments compared to the breakthrough curve of the IRA-900-C column, indicating an initial regime with relatively faster CO_2_ uptake kinetics at the beginning followed by a later stage with a noticeable slower sorption kinetics as CO_2_ loading in IRA-900-C-g increases. We surmise that the larger external surface area of IRA-900-C-g results in faster CO_2_ uptake kinetics at the initial stage of the breakthrough experiment. As more CO_2_ is adsorbed in IRA-900-C-g, water desorption occurs faster and the resin particle contracts to a greater extent (*vide infra*), resulting in the slower CO_2_ adsorption kinetics at the later stage of the experiment. Interestingly, no obvious differences in CO_2_ desorption kinetics were observed for IRA-900-C and IRA-900-C-g (Figure S9d), suggesting that the factors independent of particle sizes such as CO_2_ diffusion in the polymer bulk and reaction kinetics (step C and D in Figure 4a) play significant roles in affecting the overall CO_2_ sorption kinetics. A detailed modeling study based on breakthrough experiments or other techniques such as zero column length chromatography (ZLC) and frequency response method will be required to fully elucidate the relative contributions of each mass transfer component to the overall CO_2_ mass transfer performance.^32,34,35^

### 3.4 H_2_O Adsorption in IRA-900-C

**Figure 5:**
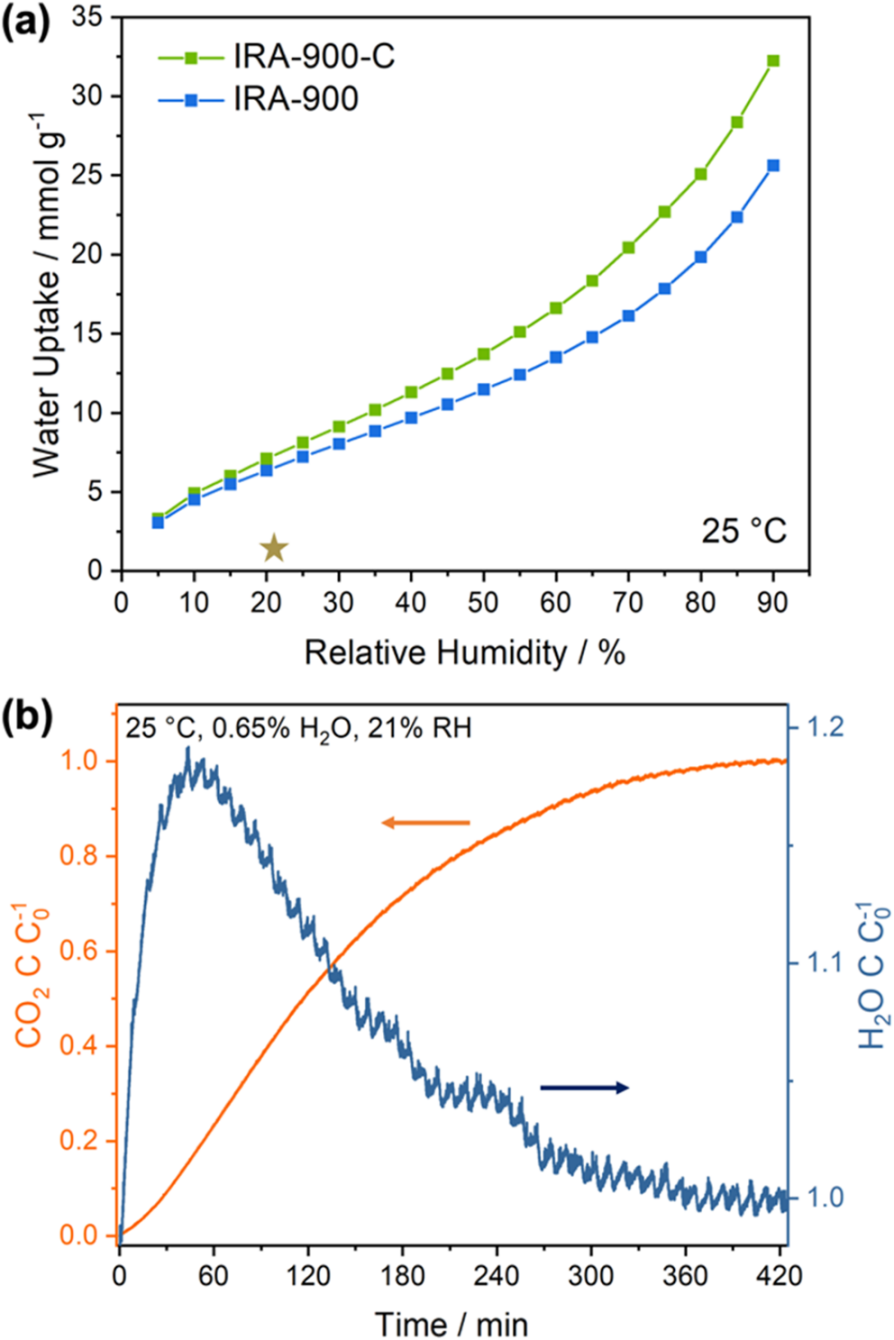
(a) Water vapor adsorption isotherms of IRA-900 and IRA-900-C at 25 °C. The star symbol indicates the estimated uptake of water vapor in IRA-900-C after the breakthrough experiment of 400 ppm CO_2_/balance N_2_. (b) CO_2_ and H_2_O breakthrough profiles of the IRA-900-C packed bed using 400 ppm CO_2_/balance N_2_ with 0.65 mol % H_2_O (21% RH) at 25 °C. The IRA-900-C bed was pre-equilibrated with N_2_ carrying 0.65 mol % H_2_O (21% RH) at 25 °C before the breakthrough experiment. The dry mass of IRA-900-C in the bed was 237.3 mg.

Water management is a critical challenge for the DAC processes. It is crucial to understand the water sorption behavior in MSA sorbents so that a judicious water management strategy can be employed for optimal DAC capacities and levelized carbon capture cost.^36−38^ Unary pseudo-equilibrium water uptakes in IRA-900 and IRA-900-C were measured gravimetrically at 25 °C and shown in Figure 5a. Water uptakes in the resins increased slowly and linearly at low RH region (RH < 45%) and rapidly increased as RH approached the saturation state (> 80%) due to water clustering around the hydrophilic QA sites. Water uptakes in IRA-900-C are slightly higher than those in the precursor under the same RH condition. We speculate that this might be attributed to the larger dimensions of HCO_3_^–^ and the hydration layer compared to Cl^–^. Water uptakes at 20 and 85% RH were 7.1 and 28.4 mmol g^–1^, respectively. It is noteworthy that water uptakes in the resin in the DAC processes could possibly deviate significantly from the unary pseudo-equilibrium uptakes because of the competitive adsorption of CO_2_ with water vapor, which has been observed in other CO_2_ adsorbents.^39−43^ Determining the coadsorption capacities of CO_2_ and H_2_O in MSA sorbents with gravimetric methods is nonideal because it is challenging to deconvolute the contribution of CO_2_ and H_2_O sorption to the weight changes of samples.^15,22^ Prior studies have assumed that the adsorption/desorption of water vapor as a result of changing RHs in the constant-volume systems is the primary reason for the sample weight changes.^5,6,14^ In contrast, breakthrough experiments allow the simultaneous calculation of the sorption capacities of different adsorbates by integrating the corresponding breakthrough curves. In order to determine the water coadsorption capacity in IRA-900-C during CO_2_ adsorption, breakthrough experiments of humid 400 ppm CO_2_ were performed after the IRA-900-C bed had been pre-equilibrated to the RH of the feeding CO_2_/N_2_ (∼21% RH) at 25 °C. The CO_2_ and H_2_O breakthrough profiles are shown in Figure 5b. The water concentration roll-up at the column outlet indicates immediate water desorption along with the introduction of CO_2_ into the bed. Water concentration gradually equilibrated to the feed concentration at the end of the breakthrough experiment, suggesting that CO_2_ adsorption leads to water desorption from the resin. Integrating the water breakthrough curves of three repeated breakthrough experiments yields an average desorbed water capacity of −5.7 mmol g^–1^, which along with the unary water uptake at 20% RH yields the coadsorbed water uptake of around 1.4 mmol g^–1^ under this condition. The CO_2_:H_2_O coadsorption ratio of 1.34 in IRA-900-C is substantially higher than those of typical DAC sorbents such as supported amines, which usually have noticeably higher H_2_O uptakes (a CO_2_:H_2_O coadsorption ratio of less than 0.2 is not uncommon, depending on the sorbent properties and RHs of DAC conditions).^36^

### 3.5 CO_2_ Desorption from IRA-900-C

**Figure 6:**
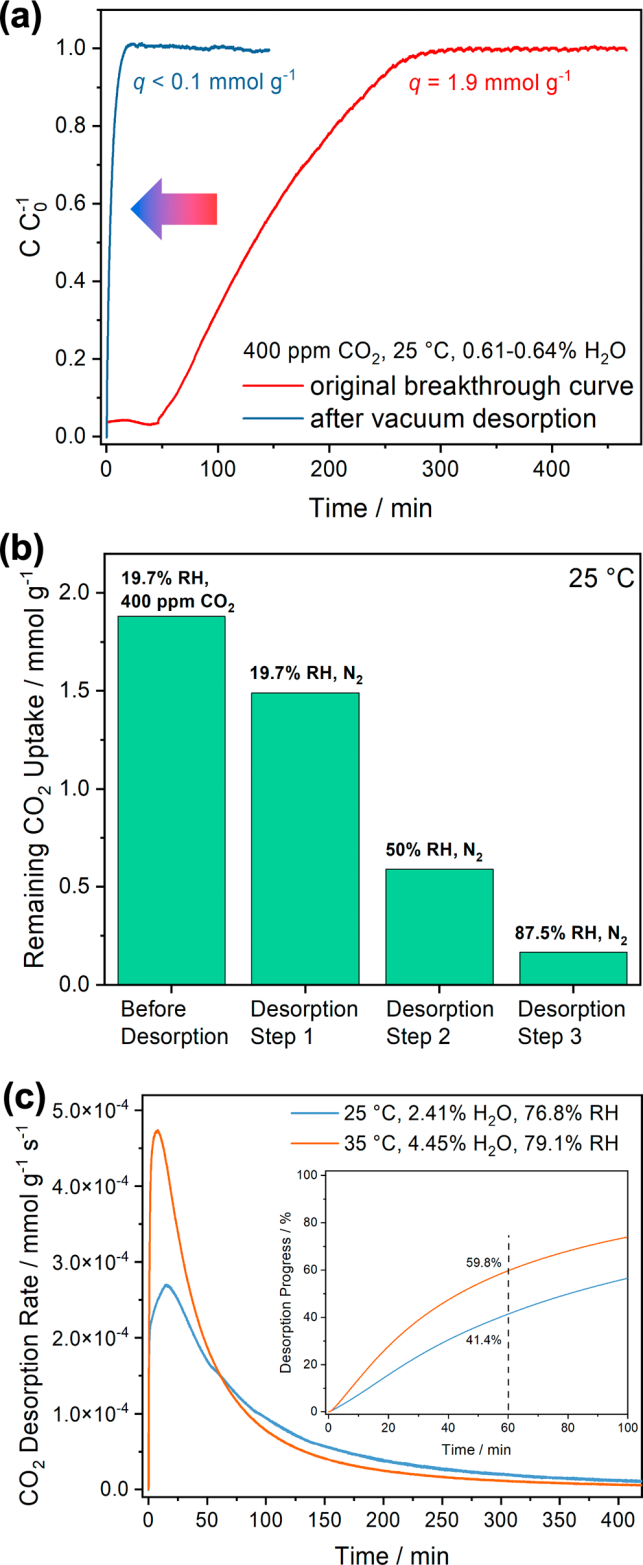
(a) Comparison of the original CO_2_ breakthrough curve and the breakthrough curve after regeneration of the IRA-900-C packed bed under a vacuum. The dry mass of IRA-900-C in the bed was 237.3 mg. (b) Remaining CO_2_ uptake capacities in IRA-900-C after desorption by N_2_ purging with different RHs. (c) Rate of CO_2_ desorption from the packed bed of IRA-900-C under humid N_2_ flows at 25 and 35 °C. The inset shows the progress of CO_2_ desorption at these two temperatures.

Understanding the CO_2_ desorption conditions is essential for designing practical DAC processes using MSA adsorbents. Dry N_2_ purging has been demonstrated as an ineffective method to activate/regenerate IRA-900-C as discussed earlier in this work (Figure 2). Further efforts to evacuate the bed using a rotary vane oil pump at room temperature could not recover the CO_2_ capture capability of IRA-900-C either (Figure 6a). These results suggest the crucial role of water vapor in triggering the desorption of CO_2_ from the resin. A series of desorption experiments was conducted by progressively varying the RH of the purging gas to investigate different CO_2_ desorption conditions. As shown in Figure 6b, 0.39 mmol g^–1^ of CO_2_ can be desorbed by switching 400 ppm CO_2_ to pure N_2_ without changing the water vapor content. This portion of desorbed CO_2_ might originate from physically adsorbed CO_2_ and part of the chemically adsorbed counterpart when the CO_2_ partial pressure decreases. Additional 0.88 and 0.41 mmol g^–1^ of CO_2_ were desorbed from the bed when the RH increased to 50 and 87.5%, respectively. These desorption experiments collectively indicate that a theoretical maximum CO_2_ working capacity of 1.27 mmol g^–1^ can be achieved with 50% RH at 25 °C and the timely removal of desorbed CO_2_ from the system. The discrepancy between the total CO_2_ desorption capacities and the adsorption capacities calculated from breakthrough experiments could be attributed to the accumulated integration errors for the lengthy desorption experiments. Figure 6c shows the CO_2_ desorption profiles of the IRA-900-C packed bed using high RH (> 76%) N_2_ flows at 25 and 35 °C immediately after breakthrough experiments. Increasing the desorption temperature enhances the maximum CO_2_ desorption rate by 75% under similar RH conditions, and 44% more CO_2_ can be desorbed from the bed within 1 h of the desorption experiments. These properties of the desorption of CO_2_ from IRA-900-C resemble other typical DAC systems based on amine adsorbents and reflect the endothermic nature of the desorption of CO_2_.

### 3.6 CO_2_ Production in Vacuum

**Figure 7:**
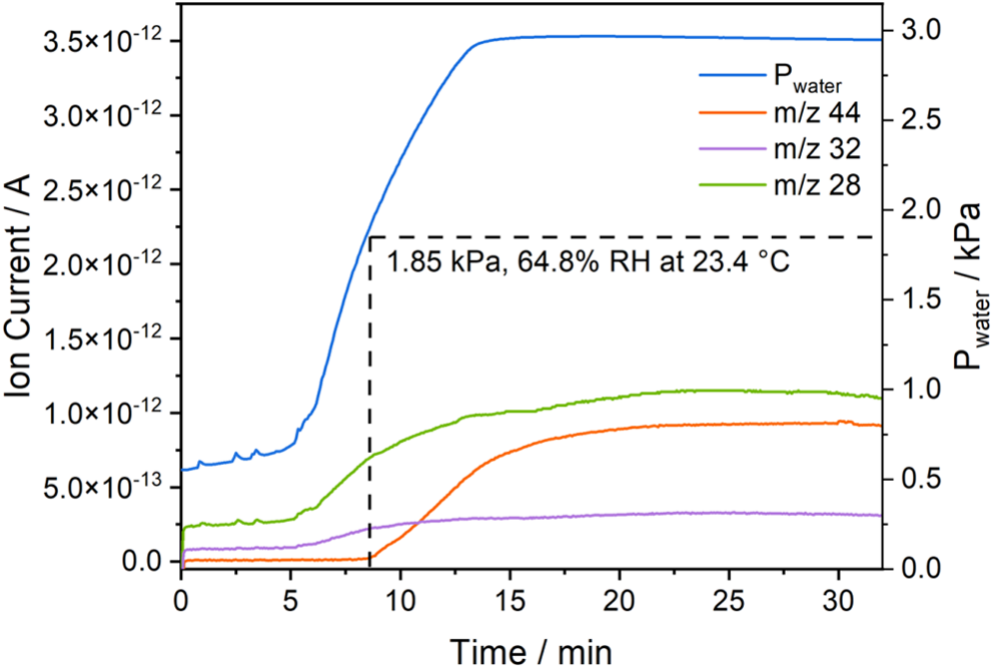
Mass spectrometer ion current profiles of different species during the CO_2_ desorption experiments in a vacuum using a packed bed of IRA-900-C.

Most works on DAC physisorbents, including the present study, investigate the behavior of CO_2_ desorption triggered by temperature or moisture swings in a dynamic (e.g., sweeping gas through the bed) or static inert atmosphere. Although these CO_2_ desorption experiments provide useful information about CO_2_ desorption capacities and kinetics, such CO_2_ desorption conditions are impractical for real-world DAC operations as the introduction of inert gases compromises the purity of CO_2_ produced from the processes. CO_2_ production in a vacuum is desirable as it eliminates the usage of sweep gases such as steam or N_2_ used in the desorption step, but relevant experiments have been rarely reported by prior works on MSA sorbents. Therefore, a closed volumetric setup shown in Scheme 3 was designed and constructed to study how water vapor stimulates CO_2_ desorption from IRA-900-C (saturated by 400 ppm CO_2_ at 25 °C and 20% RH) in a vacuum under forced convection. Air in the system was evacuated by the roughing vacuum pump, and the dissolved gas in water was also removed by freeze–pump–thaw cycles before increasing the water partial pressure in the system by heating up the water reservoir. A diaphragm circulation pump was deployed in the loop to deliver water vapor from the headspace of the water reservoir to the IRA-900-C bed. As shown in Figure 7, the mass spectrometer ion currents of mass-charge ratios relevant to CO_2_, O_2_, and N_2_, respectively, remained at low levels when water partial pressures were lower than 0.7 kPa (<5 min after the start of the experiment). As heating of the water reservoir was initiated, while other parts of the system remain at ambient temperature (Figure S10a), water partial pressure increased sharply with greater ion currents of mass-charge ratios of 32, 28, and 14 (Figures 7 and S10b), which suggests the increase of N_2_ and O_2_ partially due to the release of trapped gas molecules from the swelling resin beads. The CO_2_ ion current (44) did not increase until the partial pressure of water vapor reached 1.85 kPa, corresponding to 64.8% RH at ambient temperature. This observation is consistent with the breakthrough results that the CO_2_ uptake capacities in IRA-900-C decrease significantly only when the environmental humidity surpasses a certain threshold level. Although Figure 3a suggests an earlier onset of CO_2_ desorption from IRA-900-C at RH lower than 64.8%, it took time for the dehydrated IRA-900-C to adsorb water, which is likely the reason for the delayed moment of CO_2_ desorption. The CO_2_ signal quickly stabilized within 15 min, suggesting that an equilibrium between gas-phase and adsorbed CO_2_ was established at the given RH condition. The equilibrium time is of the same order of magnitude as the values reported in prior works that study MSA sorbents in closed systems.^24,33^ However, there could be a significant amount of CO_2_ still adsorbed in the sorbent as suggested by Figure 3b despite the quick CO_2_ desorption. Indeed, Figure S11 shows that more CO_2_ was desorbed from the resin when the bed was further regenerated under high RH N_2_ purging after the desorption experiments in a vacuum.

## 4 Conclusions

In summary, this work showcases the application of dynamic breakthrough experiments in the systematic investigation of IRA-900-C as an MSA adsorbent for DAC. Cyclic breakthrough experiments show that IRA-900-C possesses a robust CO_2_ uptake capacity of 1.92 mmol g^–1^ at 20% RH and 25 °C. More than 91% of OH^–^ sites are utilized for CO_2_ capture under this condition, which is noticeably superior to the typical amine efficiency of supported amine sorbents for DAC. Breakthrough experiments under different operating conditions are conducted to portray the landscape of CO_2_ uptake capacities of IRA-900-C as a function of temperatures, RHs, and CO_2_ partial pressures, confirming the negative impact of RHs on isothermal CO_2_ uptake capacities. CO_2_ uptake capacities and kinetics are also affected by the initial hydration degree of IRA-900-C. In addition, the high CO_2_ sorption capacity of 2.17 mmol g^–1^ at 1% CO_2_ concentration, 83% RH, and 25 °C suggests the necessity of maintaining low CO_2_ partial pressures during sorbent regeneration to achieve a reasonable CO_2_ working capacity. Breakthrough experiments with different gas velocities and particle sizes of IRA-900-C suggest that the CO_2_ adsorption in IRA-900-C is controlled by internal mass transfer resistances. Breakthrough experiments also reveal water desorption triggered by CO_2_ adsorption in the resin, providing valuable insights into water sorption management for DAC based on MSA adsorbents. A theoretical maximum CO_2_ working capacity of 1.27 mmol g^–1^ can be achieved with IRA-900-C under a constant purge of humid N_2_, and the feasibility of CO_2_ production in vacuum is experimentally verified.

Although we surmise that CO_2_ diffusion in the polymer bulk phase or (and) the reaction kinetics control the CO_2_ sorption kinetics in IRA-900-C, the mass transfer behaviors of CO_2_ and H_2_O are not fully understood so far with the current sets of breakthrough experiments. Future measurements using ion exchange resins with different texture properties and functionalities should be conducted, and detailed kinetic models considering flow dispersion and adsorption heats should be established to distinguish the contributions of different components of mass transfer resistance with a focus on extracting kinetic information in the polymer bulks and reaction kinetics. In addition, more efforts are necessary for mapping the H_2_O uptake capacities of MSA sorbents at different CO_2_ partial pressures, temperatures, and RHs. Last, the long-term stability of IRA-900-C is also a missing piece of the puzzle, which can significantly affect the capital costs of DAC processes based on this material.

## Supporting Information Available

Data processing of breakthrough experiments, additional characterizations of IRA-900 resins, supplementary CO_2_ breakthrough curves, and CO_2_ uptake capacities in IRA-900-C under different measurement conditions (PDF)
