# Supplementary material for: Investigation of Moisture Swing Adsorbents for Direct Air Capture by Dynamic Breakthrough Studies
Source: ACS Sustain Chem Eng. 2025 Apr 30;13(18):6554–64. doi: 10.1021/acssuschemeng.5c00227 (PMC12076544; doi:10.1021/acssuschemeng.5c00227)
Supplement: Supplementary file 1 — sc5c00227_si_001.pdf [file sc5c00227_si_001.pdf]

Supporting information for

Investigation of Moisture Swing Adsorbents for Direct Air Capture by Dynamic Breakthrough Studies

Yuxiang Wang,[a] Jinsu Kim,[a,b] João Marreiros,[a] Neel Rangnekar,[c] Yanhui Yuan,[c] JR Johnson,[c] Benjamin A. McCool,[c] Matthew J. Realff,[a] Ryan P. Lively*,[a]

a. School of Chemical & Biomolecular Engineering, Georgia Institute of Technology, 311 Ferst Drive., Atlanta, GA 30332, United States

b. Department of Petrochemical Materials, Chonnam National University, 50 Daehak-ro, Yeosu-si, 59631, Republic of Korea

c. Avnos, Inc. 360 Milltown Road., Bridgewater, NJ 08807, United States

Email: ryan.lively@chbe.gatech.edu

19 pages, 1 scheme, 12 figures, 1 table

1. Calculations of breakthrough experiments

The gas (CO_2 or moisture) uptake capacities, q_i, were calculated based on the breakthrough curves using the following equation.

$$q_i = \frac{p\dot{V}_0 y_{0,i}}{mRT}\int\left(1 - \frac{y_{t,i}\dot{V}_t}{y_{0,i}\dot{V}_0}\right)dt \qquad \text{Equation S1}$$

In this equation, p is the bed pressure in Pa (pressure drop was negligible with short beds), $\dot{V}_0$ is the initial (equilibrium) volumetric follow rate in m^3 s^{-1}, $\dot{V}_t$ is the volumetric follow rate at moment t in m^3 s^{-1}, $y_{0,i}$ is the composition of gas species i in the feeding gas stream, R is the gas constant, T is temperature in K, m is the sorbent mass in g, and $y_{t,i}$ is the mol fraction of gas species i recorded by the gas composition analyzer LI-850-1 at the moment t. According to the flow rates measured by the LICOR during CO_2 breakthrough experiments, the flow rate usually increases sharply after the breakthrough experiments start and fluctuates around the equilibrium value. Therefore, Equation S1 is reduced to

$$q_i' = \frac{p\dot{V}_0 y_{0,i}}{mRT}\int\left(1 - \frac{y_{t,i}}{y_{0,i}}\right)dt \qquad \text{Equation S2}$$

In the breakthrough experiments to determine H_2O coadsorption capacities, the integration of H_2O breakthrough curves yields negative values and hence negative q_{H2O}. This is because the bed of IRA-900-C was pre-equilibrated with H_2O before the breakthrough experiments, and CO_2 adsorption in IRA-900-C triggered H_2O desorption.

2. CO_2 desorption by humid N_2 purging

A typical procedure of CO_2 desorption experiments using humid N_2 purge is described as follows. After the 400 ppm CO_2 breakthrough experiments at 20% RH and 25 °C, the bed of IRA-900-C was first purged by 100 sccm N_2 with 20% RH humidity to desorb the physiosorbed CO_2

by the driving force of reduced CO_2 partial pressure. When the CO_2 concentration at the bed outlet approached equilibrium, the humidity in N_2 was progressively increased to desorb chemically bonded CO_2 from the bed. The flow rate of the purge gas only minimally changed during the desorption experiments (Figure S12). The desorbed CO_2 capacity of each regeneration step, q_d, was calculated using the following equation.

$$q_d = \frac{p\dot{V}}{mRT}\int y_t\,dt \qquad \text{Equation S3}$$

In this equation, p is the bed pressure in Pa, $\dot{V}$ is the volumetric follow rate of N_2 purge in m^3 s^{-1}, y_t is the mol fraction of CO_2 recorded by LI-850-1, R is the gas constant, T is temperature in K, and m is the sorbent mass in g.

CO_2 desorption rate in a typical CO_2 desorption experiment using humid N_2 purge was calculated using the following equation.

$$\frac{dq_d}{dt} = \frac{p\dot{V}y_t}{mRT} \qquad \text{Equation S4}$$

3. Effects of axial dispersion and adsorption heat on the dispersion of CO_2 breakthrough curves

The Peclet numbers for the experiments were calculated using different linear velocities with the molecular diffusivity of CO_2 in N_2 (0.224 cm^2 s^{-1}),[1] the bed porosity (0.4), the bed length (3.2 cm), and the particle size (about 0.06 cm according to Figure S2).[2] For the experiment with a linear velocity of 68 cm s^{-1}, the Peclet number is

$$Pe_1 = \frac{v_0 L}{D_L} = \frac{uL}{\varepsilon_b(0.7D_m + 0.5\frac{u}{\varepsilon_b}d_p)} = \frac{68\times 3.2}{0.4\times(0.7\times 0.224 + 0.5\times\frac{68}{0.4}\times 0.06)} = 103.5$$

Similarly, the Peclet number for the experiment with a linear velocity of 10 cm s^{-1} is

$$Pe_2 = \frac{v_0 L}{D_L} = \frac{uL}{\varepsilon_b(0.7D_m + 0.5\frac{u}{\varepsilon_b}d_p)} = \frac{10\times 3.2}{0.4\times(0.7\times 0.224 + 0.5\times\frac{10}{0.4}\times 0.06)} = 88.2$$

Both Peclet numbers are significantly greater than 1, suggesting that axial dispersion should have minor contribution to the mass transfer of CO_2 under these experimental conditions

The temperature of the packed bed is unlikely to change significantly as a result of CO_2 adsorption because of the dilute CO_2 concentration. Assuming that the CO_2 heat of adsorption is as high as −90 kJ mol^{-1} and all CO_2 is adsorbed by the bed within the first second of the breakthrough experiment using 200 sccm of 400 ppm CO_2 flow, the released adsorption heat is

$$\frac{0.2}{22.4}\frac{mol}{min}\times\frac{1}{60}\frac{min}{s}\times\frac{400}{1000000}\times 90\frac{kJ}{mol}\times\frac{1000}{1}\frac{J}{kJ}\times 1s = 0.0054\,J$$

This amount of energy will be dissipated quickly by the bulk flow, leading to negligible temperature increase in the bed.

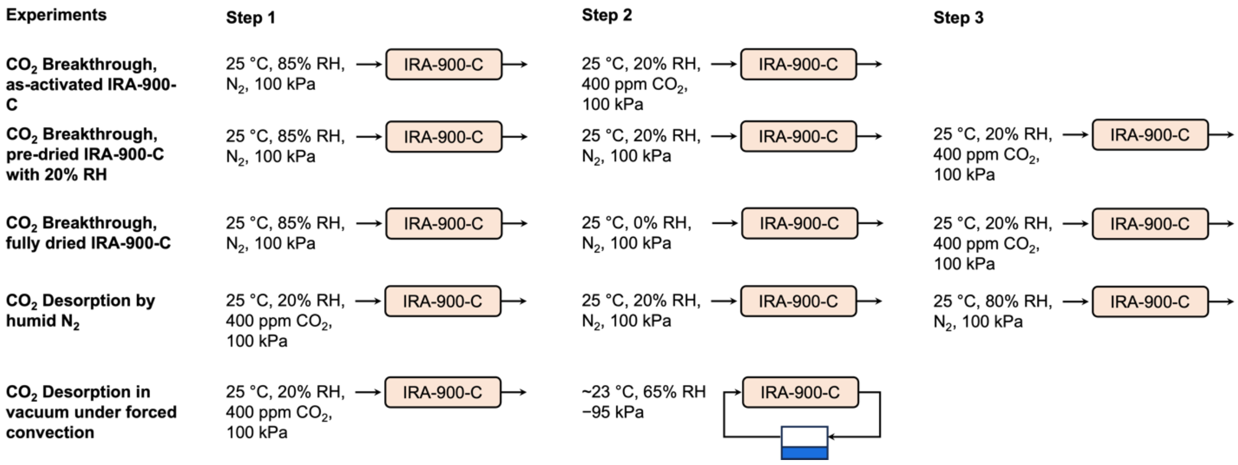

Scheme S1. Summary of experimental conditions of representative CO_2 adsorption and desorption experiments discussed in this study.

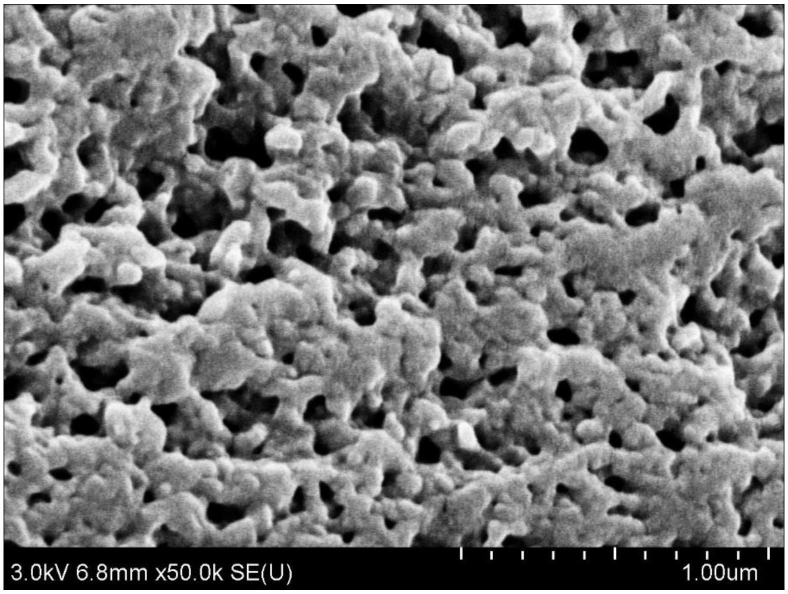

Figure S1. A scanning electron microscopy (SEM) image of the surface of IRA-900 before anion exchange.

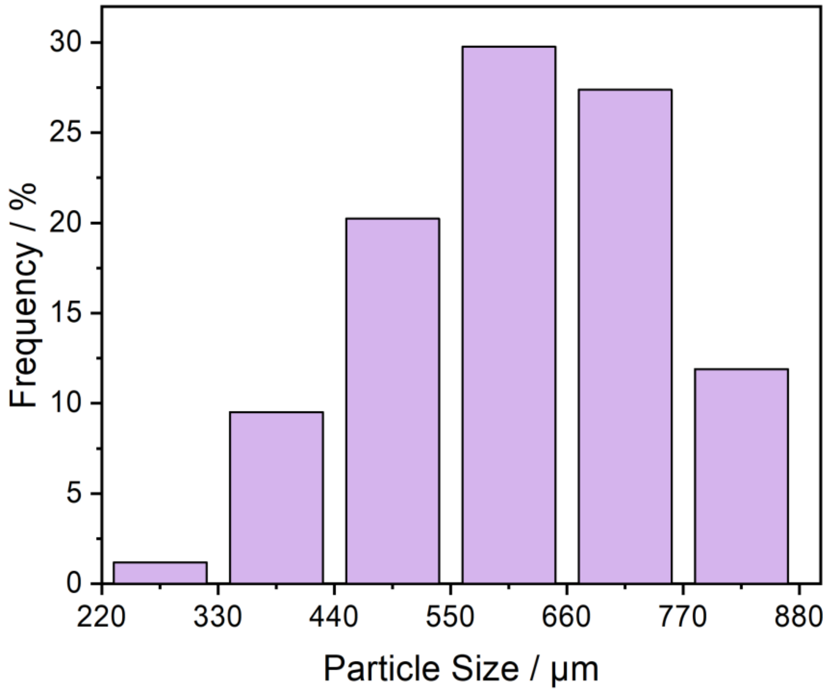

Figure S2. Particle size distribution of IRA-900-C.

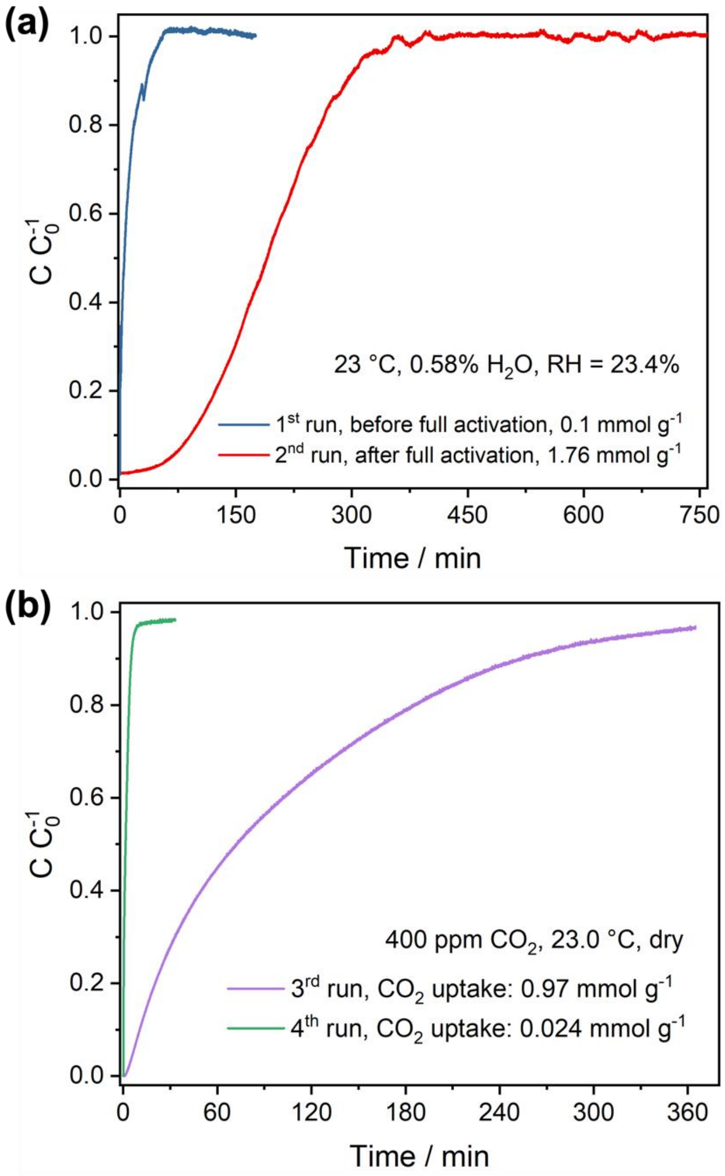

Figure S3. (a) The first two CO_2 breakthrough curves of the IRA-900-C packed bed before and after full activation. These curves were obtained using 400 ppm CO_2 / balance N_2 with 0.58 mol% H_2O (23.4% RH) at 23 °C. (b) CO_2 breakthrough curves of the IRA-900-C packed bed obtained with dry 400 ppm CO_2 / balance N_2 at 23 °C. Prior to the 3rd breakthrough run, the bed was fully regenerated by humid Ar (RH > 80%) after the 2nd breakthrough run. The bed was purged by dry Ar overnight after the 3rd breakthrough run and before starting the 4th breakthrough experiment. The mass of dry IRA-900-C in the bed was 357.0 mg.

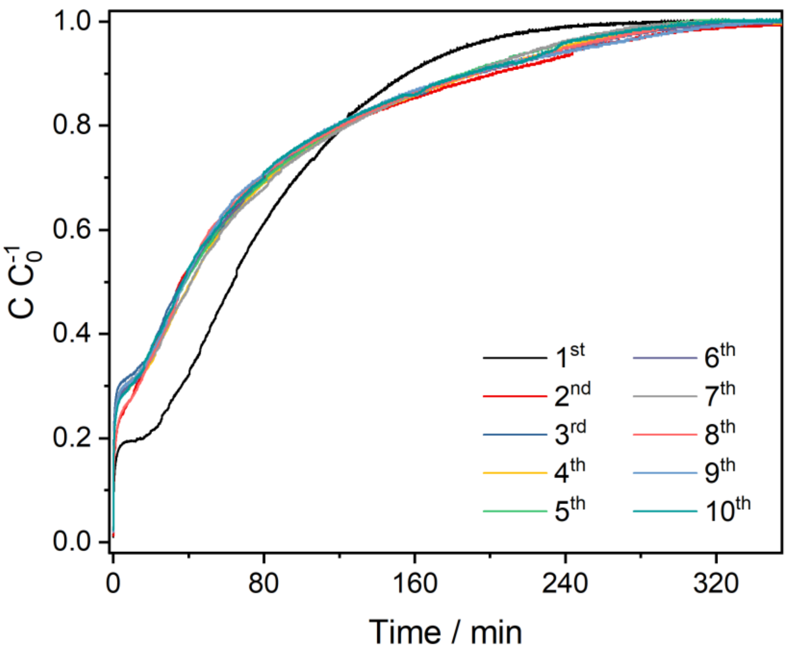

Figure S4. The CO_2 breakthrough curves of the repeated breakthrough experiments using a packed bed of IRA-900-C and 400 ppm CO_2 at 25 °C and 21% RH. The mass of dry IRA-900-C in the bed was 115.6 mg.

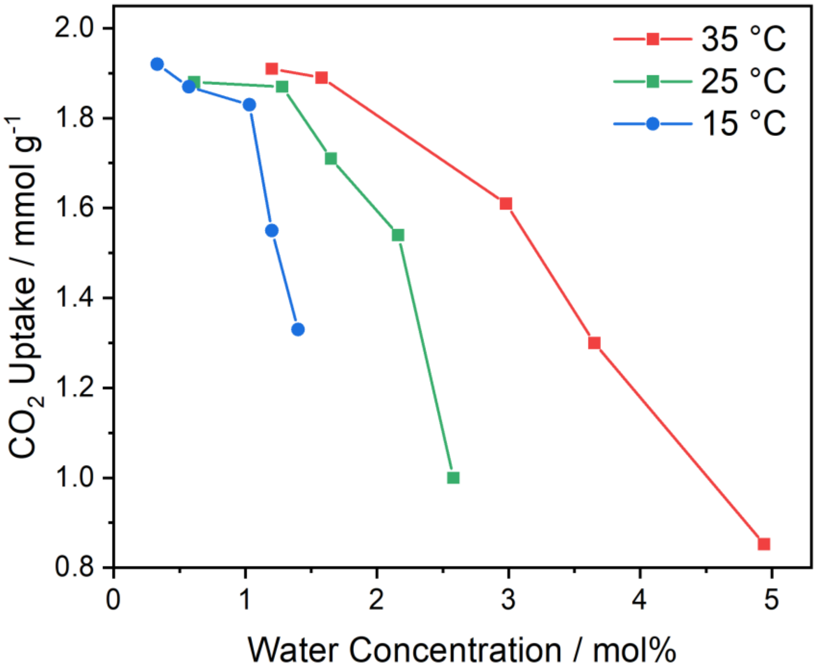

Figure S5. CO_2 adsorption capacities in IRA-900-C as a function of mol fractions of water vapor at 15, 25, and 35 °C.

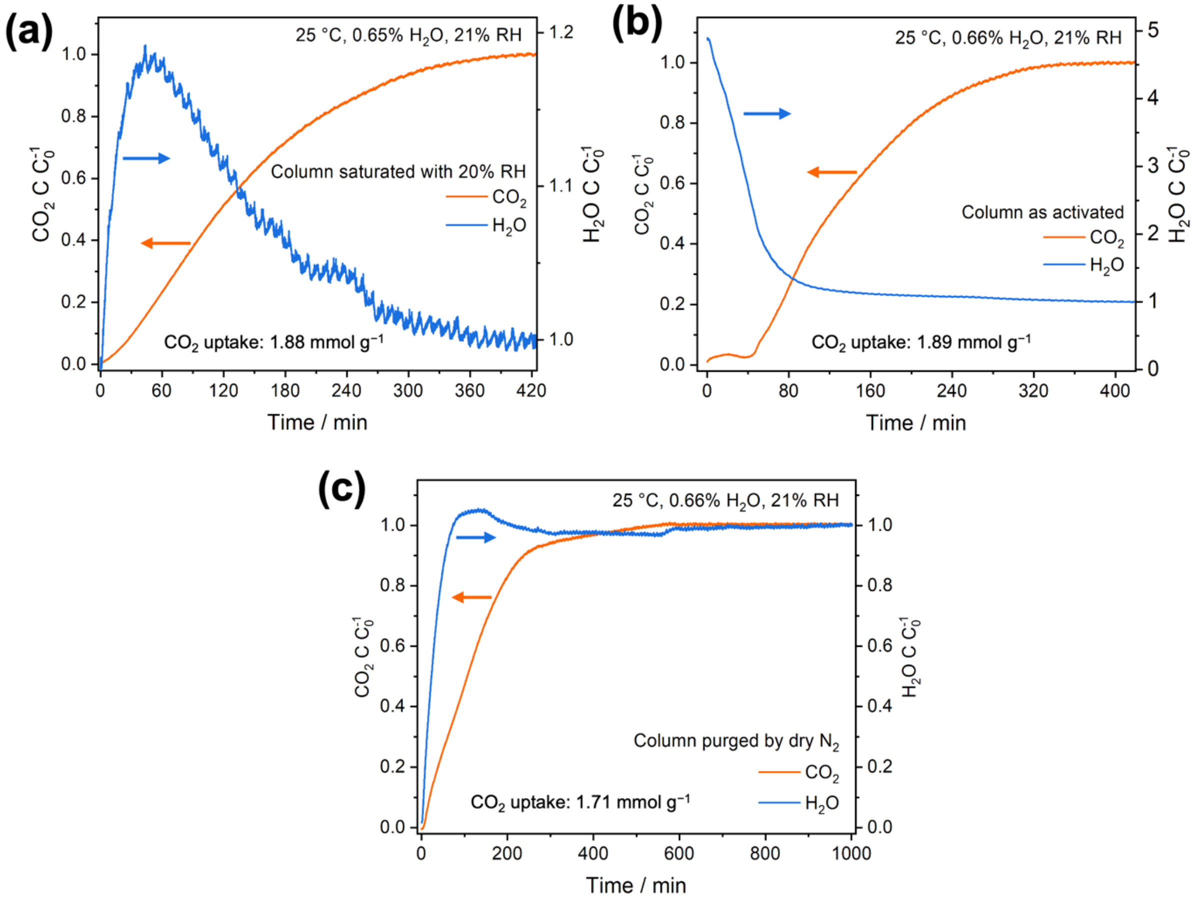

Figure S6. Comparison of CO_2 and H_2O breakthrough curves of the IRA-900-C packed bed with different initial conditions, namely, (a) pre-dried with 20% RH moisture, (b) as activated, and (c) purged by dry N_2 overnight, before the breakthrough experiments. The experiments were obtained with 400 ppm CO_2 / balance N_2 with 0.67 mol% H_2O (21% RH) at 25 °C. The mass of dry IRA-900-C in the bed was 237.3 mg.

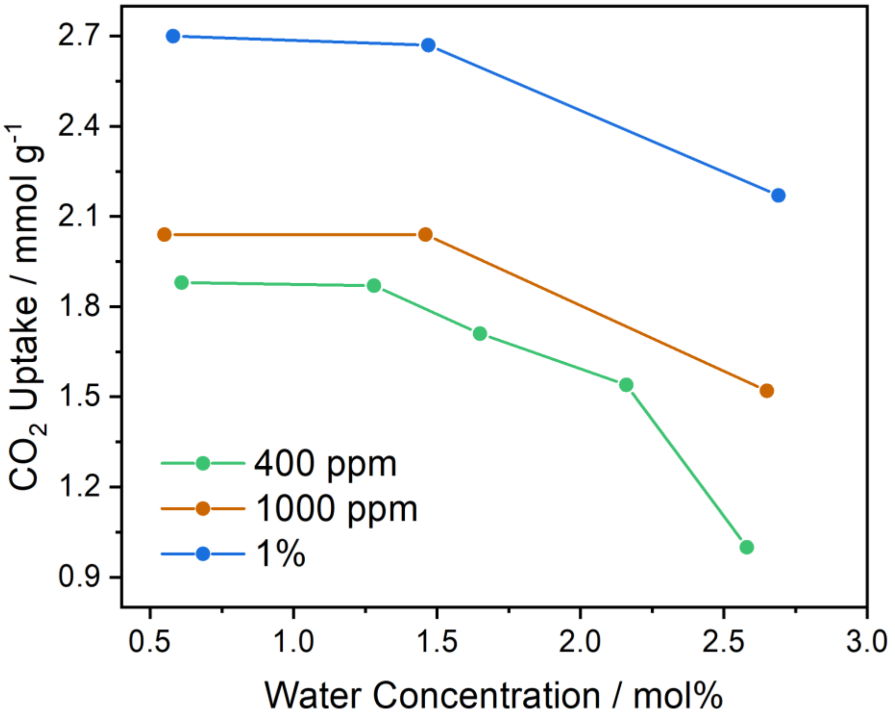

Figure S7. CO_2 uptake capacities of IRA-900-C at different humidity levels and different CO_2 concentrations at 25 °C.

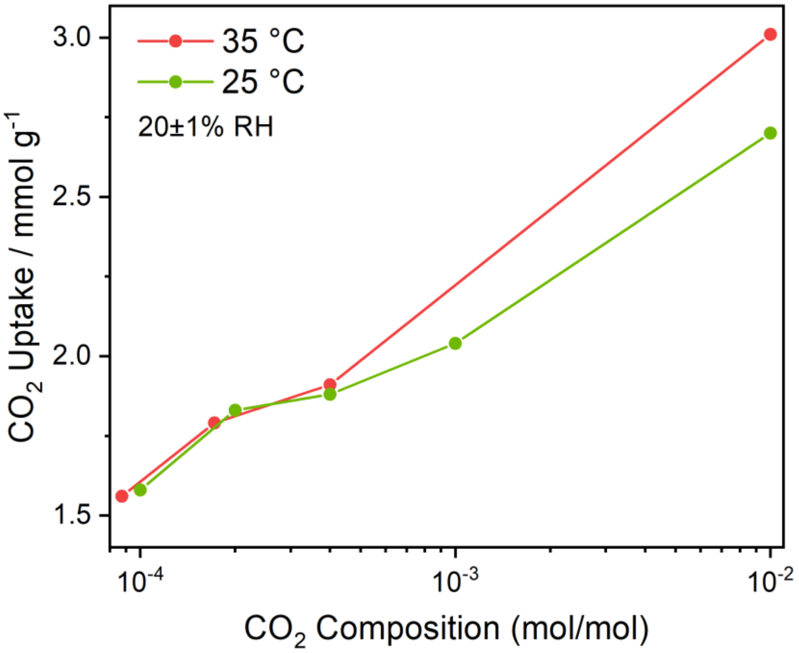

Figure S8. CO_2 uptake capacities of IRA-900-C at different CO_2 concentrations in the presence of 20±1% RH at 25 and 35 °C.

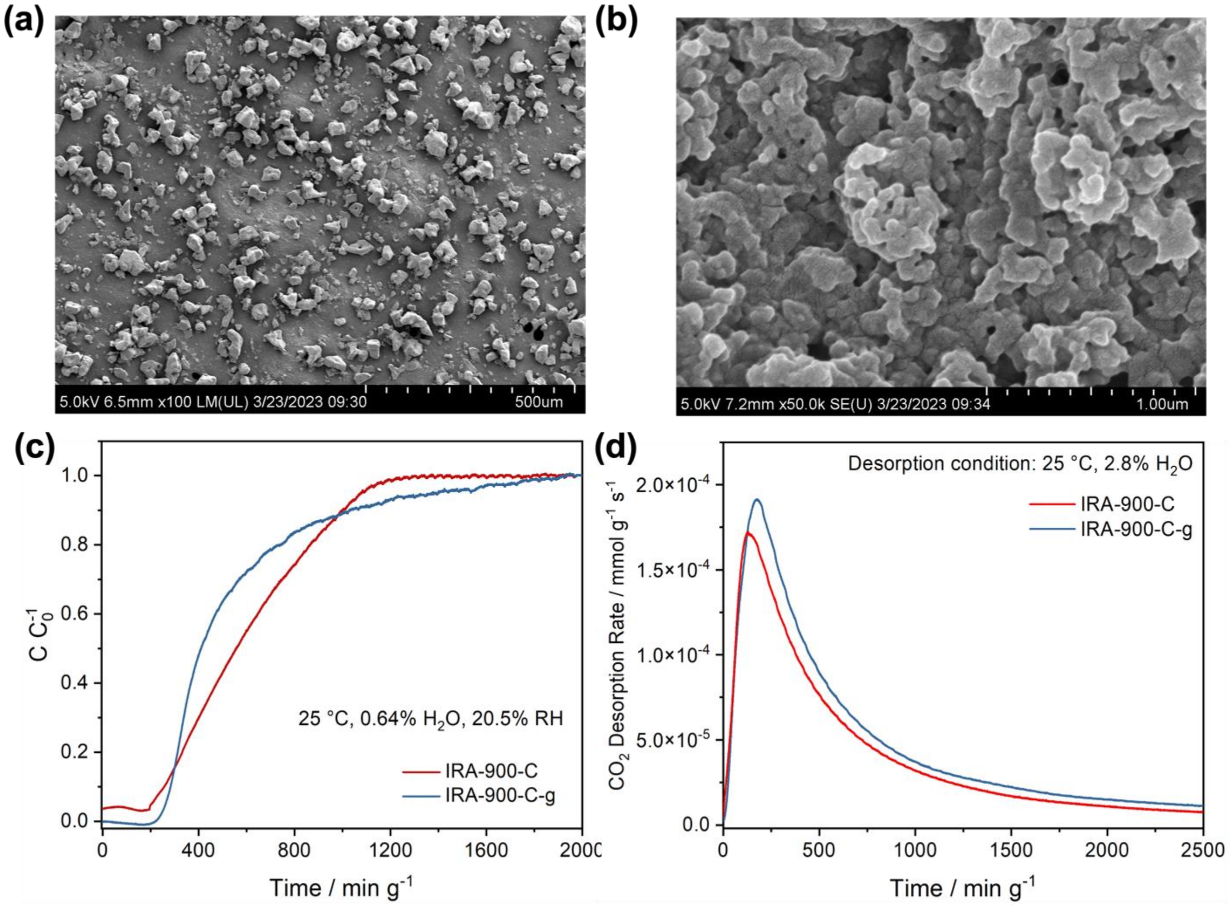

Figure S9. (a) SEM image of IRA-900-C-g. (b) Close-up SEM image of IRA-900-C-g showing the macroporous texture on the surface of the sorbent. (c) The CO_2 breakthrough curves of IRA-900-C and IRA-900-C-g beds using 400 ppm CO_2 / balance N_2 with 0.64% H_2O at 25 °C. (d) The rate of CO_2 desorption from the packed beds of IRA-900-C and IRA-900-C-g under humid N_2 flows (~ 89% RH) at 25 °C. The desorption curves were obtained after purging the beds with dry (~ 20.5% RH) N_2 after the breakthrough experiments until CO_2 signals detected at the bed exit were stable.

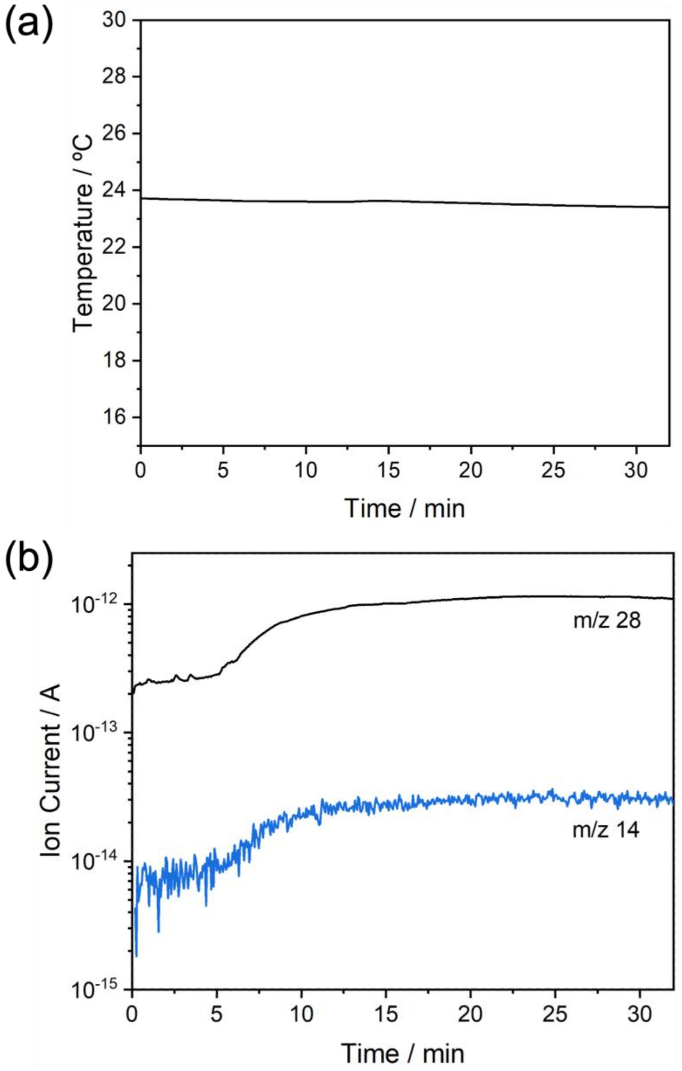

Figure S10. (a) Temperature profile recorded by the humidity detector during the CO_2 desorption experiment under vacuum. (b) Mass spectrometer ion currents of mass-charge ratios of 28 and 14 during the CO_2 desorption experiment in vacuum.

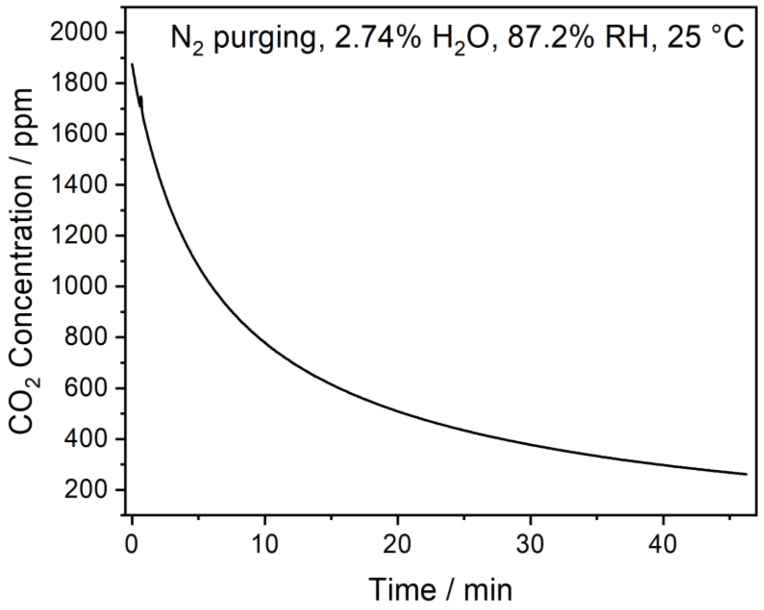

Figure S11. The CO_2 concentration at the outlet of the partially regenerated IRA-900-C packed bed under the purging of humid N_2 (87.2% RH) at 25 °C.

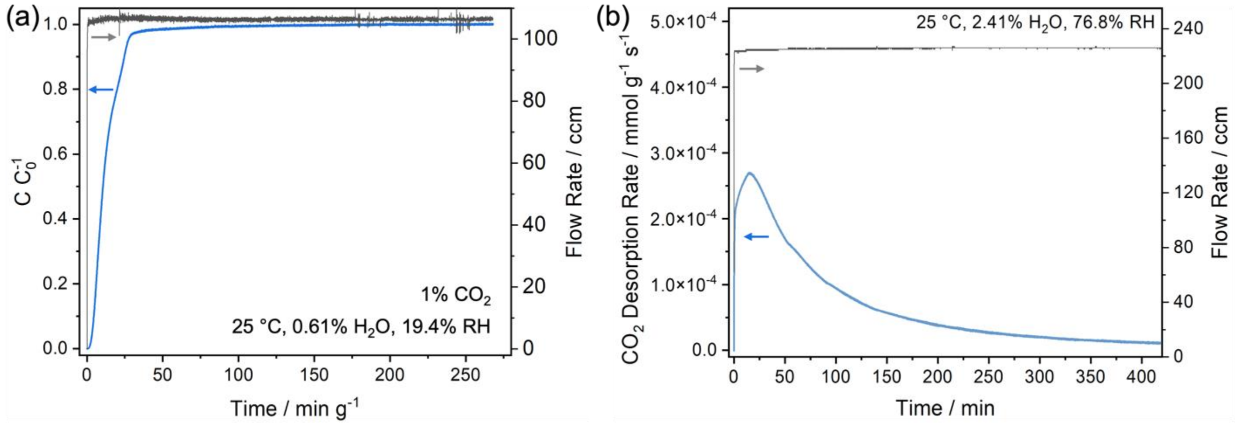

Figure S12. (a) CO_2 breakthrough curve and effluent flow rate during a breakthrough experiment using 1% CO_2 with the IRA-900-C packed bed. (b) The effluent flow rate and rate of CO_2 desorption from the IRA-900-C packed bed (saturated by 400 ppm CO_2) under humid N_2 flow at 25 °C.

Table S1. Elemental compositions of IRA-900 and IRA-900-C.

Resins	C / wt%	H / wt%	N / wt%	Cl / wt%	O / wt% *
IRA-900	62.65 ± 0.10	8.66 ± 0.09	5.90 ± 0.02	14.76 ± 0.10	~ 8.03
IRA-900-C	58.08 ± 0.12	8.28 ± 0.09	5.01 ± 0.02	< 0.25	> 28.38

* The contents of O are estimated based on the compositions of other elements.

4. References

(1) *Self-Diffusion and Binary-Diffusion Coefficients in Gases, Technical Note*, https://doi.org/10.6028/NIST.TN.2279, Mar 2nd, 2025.
(2) Farooq, S.; Qinglin, H.; Karimi, I. A. Identification of Transport Mechanism in Adsorbent Micropores from Column Dynamics. *Ind. Eng. Chem. Res.* **2002**, *41*, 1098-1106. 10.1021/ie0104621
